# Integrative Single‐Cell and Spatial Transcriptomics Reveals the Crosstalk of CTHRC1+ CAF and MMP7+ Epithelial Axis as a Potential Therapeutic Target and Predicts Poor Clinical Outcomes in Colorectal Cancer

**DOI:** 10.1155/mi/9314553

**Published:** 2026-04-08

**Authors:** Yang Yang, Song Huang, Hanyu Zhou, Yingjian Wang, Sisi Wu

**Affiliations:** ^1^ Department of Gastroenterology, Taizhou Second People’s Hospital Affiliated to Yangzhou University, Taizhou, Jiangsu, China; ^2^ Department of Anorectal Surgery, Fenggang County Traditional Chinese Medicine Hospital, Zunyi, Guizhou, China; ^3^ Department of Oncology, The Third Affiliated Hospital of Soochow University, Changzhou, Jiangsu, China, suda.edu.cn; ^4^ The First Clinical Medical College of Zhengzhou University, Zhengzhou, Henan, China; ^5^ Department of Ultrasound, Taizhou Second People’s Hospital Affiliated to Yangzhou University, Taizhou, Jiangsu, China

**Keywords:** cancer-associated fibroblasts, colorectal cancer, immunosuppressive niche, single-cell resolution analysis, tumor microenvironment

## Abstract

**Background:**

Colorectal cancer (CRC) progression is heavily influenced by the tumor microenvironment (TME), where cancer‐associated fibroblasts (CAFs) are key players. However, the heterogeneity, plasticity, and functional roles of CAFs in CRC remain poorly understood.

**Methods:**

We integrated single‐cell RNA sequencing (scRNA‐seq) data from four public CRC datasets and spatial transcriptomics data. Using computational approaches such as Harmony, Monocle2, and CellChat algorithms, we analyzed cellular landscapes, CAF subtype identification, developmental trajectories, transcription factor networks, and cell–cell communications to reveal CAF heterogeneity and their crosstalk with other cell subtypes in CRC.

**Results:**

We identified eight distinct CAF subtypes with unique gene expression profiles and developmental plasticity. The CTHRC1+ CAF subtype was significantly associated with T cell exclusion and upregulated expression of immune checkpoint genes. We uncovered a specific communication axis between CTHRC1+ CAFs and MMP7+ malignant epithelial (Malig‐Epi) cells mediated by the thrombospondin (THBS)2‐SDC4 ligand–receptor signaling. High infiltration of both cell types synergistically correlates with worse prognosis and unfavorable response to immunotherapy.

**Conclusions:**

Our study delineates CAF heterogeneity in CRC and highlights the CTHRC1+ CAF subtype as a critical organizer of an immunosuppressive niche. The THBS2‐SDC4 signaling pathway between CTHRC1+ CAFs and MMP7+ epithelial cells acts as a potential therapeutic target to disrupt protumorigenic crosstalk and improve clinical outcomes for CRC patients.

## 1. Introduction

Colorectal cancer (CRC) remains one of the most prevalent and lethal malignancies worldwide, posing significant challenges in early detection and effective treatment [1–3]. The tumor microenvironment (TME) plays a pivotal role in CRC progression, influencing tumor growth, metastasis, and response to therapies [4–6]. Within this complex ecosystem, cancer‐associated fibroblasts (CAFs) have emerged as a critical component, contributing to the structural and functional heterogeneity of the TME [7–9]. These cells exhibit remarkable plasticity and interact dynamically with other cellular constituents, including epithelial cells, immune cells, and the extracellular matrix (ECM), thereby modulating tumor behavior and therapeutic resistance [10–12].

Recent advances in single‐cell RNA sequencing (scRNA‐seq) and spatial transcriptomics have revolutionized our understanding of the cellular and molecular landscapes within CRC [13, 14]. These technologies have revealed the existence of distinct CAF subtypes, each with unique gene expression profiles and functional roles. However, the precise mechanisms underlying CAF heterogeneity and its impact on CRC progression and therapeutic outcomes remain incompletely understood. Elucidating these mechanisms is crucial for developing targeted therapies that can effectively modulate the TME and enhance treatment efficacy.

We aim to comprehensively characterize the heterogeneity and plasticity of CAFs in CRC, investigate their interactions with other cellular components, and assess their clinical relevance. By integrating scRNA‐seq data from multiple datasets and spatial transcriptomics data, we constructed a detailed cellular landscape of CRC, identifying and annotating various CAF subtypes. We employed advanced computational approaches, pseudotime analysis, and transcription factor regulon inference to dissect the developmental trajectories and transcriptional regulatory networks of CAFs. Additionally, we performed functional enrichment analysis to identify biological pathways significantly enriched in different CAF subtypes, providing insights into their potential functions. Furthermore, we explored the interactions between CAFs and other cell types within the TME, focusing on the thrombospondin (THBS)2‐SDC4 signaling pathway, which has been implicated in fibroblast–epithelial cell communication [15, 16]. By analyzing cell–cell communication networks and ligand–receptor interactions, we aimed to uncover the role of CAFs in shaping the TME and influencing CRC progression. We also assessed the clinical significance of CAF subtypes by correlating their infiltration levels with CRC patient survival and response to immunotherapy.

Our findings reveal a complex interplay between CAFs and other cellular components in CRC cells, highlighting the role of specific CAF subtypes in promoting tumor growth and immune evasion. The identification of the THBS2‐SDC4 signaling pathway as a key mediator of CAF–epithelial cell communication provides a potential therapeutic target for disrupting the protumorigenic TME in CRC. Moreover, our analysis of CAF infiltration and its association with clinical outcomes underscores the potential of CAF subtypes as biomarkers for predicting treatment response and prognosis.

In summary, our study provides a comprehensive characterization of CAF heterogeneity and its implications in CRC, offering novel insights into the cellular and molecular mechanisms driving tumor progression and resistance to therapies. By integrating single‐cell and spatial transcriptomics data with computational modeling, we have identified key CAF subtypes and signaling pathways that may serve as targets for developing more effective CRC treatments. We hope these findings can pave the way for future research aimed at modulating the TME to improve clinical outcomes for CRC patients.

## 2. Materials and Methods

### 2.1. Data Acquisition

scRNA‐seq data for CRC were sourced from four datasets: GSE221575, GSE200997, GSE144735, and GSE132465 [17–19]. Spatial transcriptomics data were obtained from the Cancer Diversity Asia (CancerDiversity, http://www.cancerdiversity.asia/scCRLM) database [20], and two additional samples, IntestineCancer and Parent_WTA, were acquired from the 10x Genomics official website.

### 2.2. scRNA‐seq Analysis

The Seurat (v4.3.0) R package was applied for downstream analyses [21]. Low‐quality cells (UMIs 15% mitochondrial genes) were filtered out, and potential doublets were identified using DoubletFinder (v2.0.4), resulting in a comprehensive dataset comprising 140,717 cells across 71 samples. Batch effects among different samples were corrected using the Harmony integration algorithm [22]. Copy number variation (CNV) scores were estimated using the inferCNV algorithm and were compared among different cell types and subtypes.

### 2.3. Classification and Annotation of Cellular Components

Classification was achieved by leveraging the expression profiles of established marker genes. This classification scheme encompassed epithelial cells, fibroblasts, smooth muscle cells/pericytes, endothelial cells, myeloid cells, mast cells, T/NK cells, B cells, and plasma cells. The differential expression of signature genes within each cell type was analyzed to provide a molecular blueprint of the CRC microenvironment. Differentially expressed genes (DEGs) were identified with the “FindAllMarkers” function. Statistical significance was evaluated with the Wilcoxon rank‐sum test adjusted by the Benjamini–Hochberg method.

### 2.4. CAF Subtype Identification and Analysis

CAFs were categorized into eight distinct subtypes, including CTHRC1+, C7+, CCL8+, MFAP5+, ENHO+, COPS9+, MMP3+, and WT1+ fibroblasts. The proportion of each CAF subtype across different sample types was analyzed, distinguishing between tumor, border, and normal samples. The expression levels of the top DEGs across the eight CAF subtypes were displayed. A radar chart illustrated the average expression of six CAF subtype‐specific gene sets, as calculated by the AUCell algorithm. A heatmap represented the average expression of genes associated with each CAF subtype.

### 2.5. Pseudotime Analysis

The developmental trajectory of CAFs was constructed using the Monocle2 algorithm [23]. The trajectory was delineated using the top 100 DEGs that distinguish between CAF subclusters. Dimensionality reduction of the data was executed with the “reduceDimension” function using the DDRTree method. Genes exhibiting significant correlation with pseudotime were ascertained through the differentialGeneTest. The branch‐dependent transcriptional programs were generated using BEAM, and the dynamics of gene expression were visualized across pseudotime branches with plot_genes_branched_pseudotime.

### 2.6. Transcription Factor Regulon Inference

Transcriptional regulatory networks were constructed using the pySCENIC algorithm, which calculates regulon activity based on coexpression and motif enrichment [24]. Subtype‐specific transcription factors were identified by the Wilcoxon test, and regulon specificity scores were calculated using the Jensen–Shannon divergence via the “philentropy” R package.

### 2.7. Gene Set Enrichment Analysis (GSEA)

GSEA was performed to identify significantly enriched biological pathways among different CAF subtypes. The GSEA analysis compared the expression profiles of genes associated with specific biological pathways, identifying pathways that were significantly enriched in specific CAF subtypes.

### 2.8. Cell–Cell Interaction Analysis

Potential interactions between cell types were predicted using scRNA‐seq data and the CellChat software package (v2.2.0) [25]. CellChatDB.human is the reference database for ligand–receptor interactions. We employed global interaction mapping to pinpoint cell–cell communication pathways that demonstrated differential detection between tumor tissues and their adjacent noncancerous counterparts. Reciprocal signaling was identified through ligand–receptor mapping. To further characterize crosstalk between CAF subtypes and malignant epithelial (Malig‐Epi) subtypes, we employed the NicheNet package to infer ligand–target interactions [26]. Heatmaps were used to display average expression across subtypes after scaling.

### 2.9. Analyses of Survival and Therapy Outcomes

Overall survival was analyzed using the survival R package. Hazard ratios (HRs) and 95% confidence intervals (CIs) were calculated via Cox proportional hazards regression. Kaplan–Meier curves were plotted, and group comparisons were made using the log‐rank test. Response rates to immune checkpoint blockade (ICB) were compared by the chi‐square test.

### 2.10. Spatial Transcriptomics Analysis

Spatial gene‐spot matrices from ST and Visium data were analyzed with Seurat R package. Data were normalized with LogVMR. Dimensionality reduction was conducted using PCA. The BayesSpace R package was used for spatial enhancement (spatialEnhance and enhanceFeatures functions). Single‐cell‐derived signature scores were mapped to ST data using AddModuleScore in Seurat, and spatial expression was visualized with SpatialFeaturePlot. The SpaGene algorithm was applied to identify three spatial gene expression modules per sample, and interpattern similarity was computed by the Jaccard index, visualized using ComplexHeatmap R package.

### 2.11. Statistical Analysis

All statistical analyses were performed in R. Correlations were assessed using Spearman’s or Pearson’s test. Group comparisons employed Kruskal–Wallis and Wilcoxon rank‐sum tests with Benjamini–Hochberg correction for multiple testing. A *p*‐value < 0.05 was considered statistically significant.

## 3. Results

### 3.1. Comprehensive scRNA‐seq Analysis and Cellular Landscape in CRC Samples

We executed a scRNA‐seq analysis on CRC patient samples sourced from four distinct datasets: GSE221575, GSE200997, GSE144735, and GSE132465. Following stringent quality control procedures, which entailed the elimination of background noise and the removal of doublets, a comprehensive dataset comprising 140,717 cells across 71 samples was curated for further investigation. Post batch effect normalization and clustering, the dataset was stratified into nine predominant cell types, as depicted in Figure 1A. Two pie charts show the composition of sample and cell numbers in this study, with different colors representing different datasets (Figure 1B,C). Epithelial cells, fibroblasts, smooth muscle cells, endothelial cells, myeloid cells, mast cells, T/NK cells, B cells, and plasma cells were annotated with established marker genes, and the UMAP plots display the expression landscape of marker genes within each cell type, thereby providing a molecular blueprint of the CRC microenvironment (Figure 1D). Figure 1E presents a dot plot that quantifies the expression levels and prevalence of canonical marker genes across the primary cell types. This graphical representation elucidates the gene expression heterogeneity intrinsic to the diverse cellular compartments within CRC. The bar plots in Figure 1F reveal the proportional distribution of seven major cell types across various sample categories, coupled with the aggregate cellular counts for each type. These data provide insights into the cellular compositional variance among the CRC samples. Figure 1G offers a comparative assessment of cellular proportions, absolute cell counts, and transcriptome profiles across the nine identified cell types and four distinct datasets. This statistical overview accentuates the interdataset variability and consistency in cellular representation and molecular signatures.

Figure 1Comprehensive single‐cell RNA sequencing analysis reveals the cellular landscape of colorectal cancer. (A) UMAP plot shows that a total of 140,717 cells integrated from four CRC scRNA‐seq datasets (including 71 CRC samples) were clustered into nine major cell types. (B, C) Pie charts show the composition of sample and cell numbers in this study. (D) Expression of canonical marker genes across cell types. (E) Dot plot illustrating the expression level and percentage of key marker genes in each cell type. (F) Bar plot depicting the proportion of seven major cell types across different sample categories. (G) Comparative analysis of cellular composition, absolute counts, and transcriptomic profiles across nine cell types and four datasets.

**Figure:**
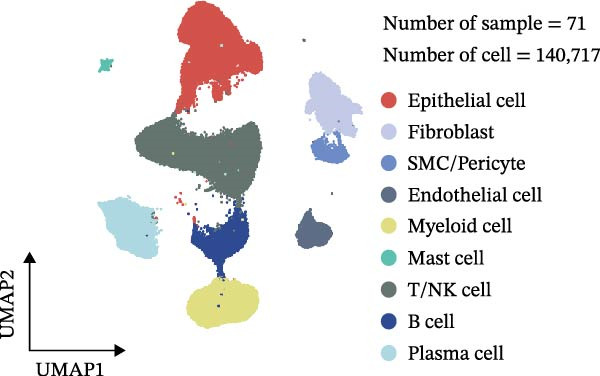
(A)

**Figure:**
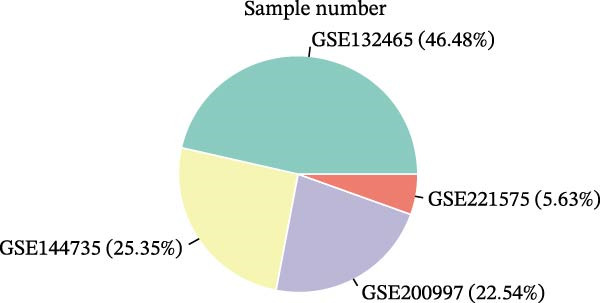
(B)

**Figure:**
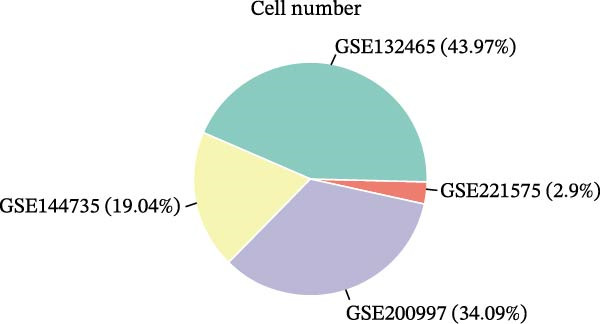
(C)

**Figure:**
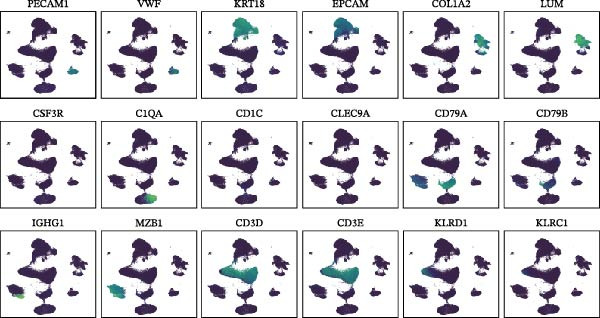
(D)

**Figure:**
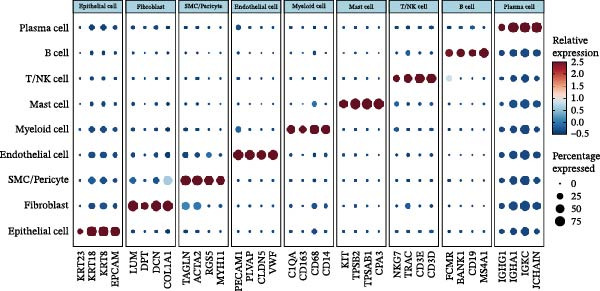
(E)

**Figure:**
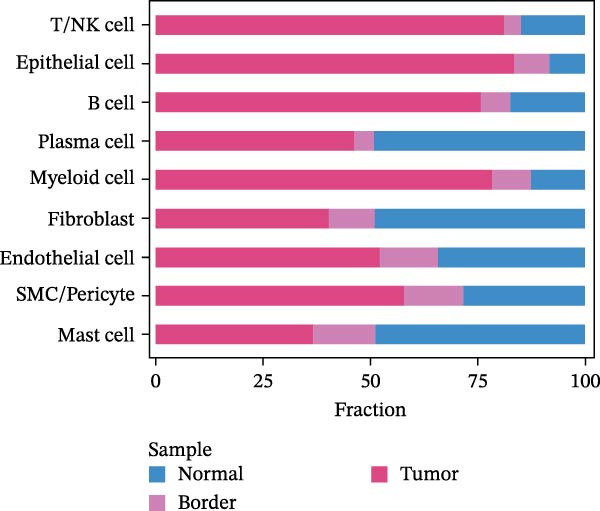
(F)

**Figure:**
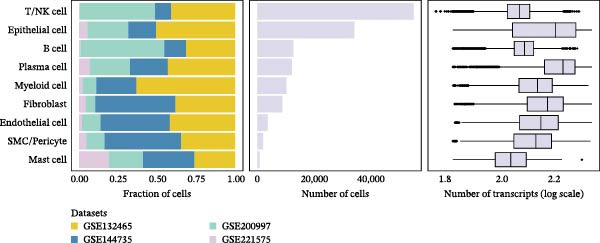
(G)

### 3.2. Heterogeneity and Plasticity of CAFs in CRC

The UMAP plot categorizes CAFs into eight distinct subtypes, including CTHRC1+, C7+, CCL8+, MFAP5+, ENHO+, COPS9+, MMP3+, and WT1+. Each subtype was represented by a unique color (Figure 2A and Figure S1A). The distribution of these CAFs in normal, border, and tumor samples (Figure S1B), and the distribution of cell cycle states among different CAF subtypes is shown in Figure S1C. A UMAP plot shows the distribution of the phenotypic scores of six established subtypes (iCAF, myCAF, apCAF, pCAF, vCAF, and lpCAF) among all CAFs identified in our study (Figure S1D). Two heatmaps show the lipoxygenase (LOX)‐, matrix metalloproteinase (MMP)‐, and collagen (COL)‐related genes in different CAF subtypes (Figure S1E,F). The stacked plot reveals the proportion of each CAF subtype across different sample types, with colors distinguishing between tumor, border, and normal samples (Figure 2B). This analysis underscores the variable distribution of CAF subtypes in distinct TMEs. A Sankey plot depicts the proportion of CAF subtypes within three sample types, with colors representing different CAF clusters (Figure 2C). This visualization highlights the differential representation of CAF subtypes across tumor, border, and normal regions. Dot plots display the expression levels of the top three DEGs across the eight CAF subtypes. The color intensity indicates the average expression value, while the dot size represents the percentage of gene expression (Figure 2D). A radar chart illustrates the average expression of six CAF subtype‐specific gene sets, as calculated by the AUCell algorithm, which provides a comprehensive overview of the transcriptional profiles associated with each CAF subtype (Figure 2E). A heatmap represents the average expression of genes associated with each CAF subtype (Figure 2F). This analysis allows for the identification of gene expression patterns that are unique to each subtype, facilitating a deeper understanding of their functional roles. Figure 2G depicts a heatmap of differential activation of pathways across CAF subtypes, indicating deviations from the overall mean, with colors representing scaled scores, highlighting pathways that are differentially activated in specific CAF subtypes. KEGG pathway enrichment analysis for DEGs across CAF subtypes is presented in a heatmap format, with colors indicating the standardized values of −log_10_ *p*‐values (Figure 2H). This analysis identifies pathways that are significantly enriched in specific CAF subtypes. Box plots demonstrate the infiltration levels of the eight CAF subtypes in tumor and normal samples from the TCGA‐COAD dataset (Figure 2I). This comparison reveals differences in the abundance of CAF subtypes between tumor and normal tissues, suggesting their potential roles in tumor progression. In summary, these analyses provide a detailed characterization of CAF heterogeneity and plasticity in CRC, identifying distinct subtypes with unique gene expression profiles and pathway activations.

Figure 2Heterogeneity and plasticity of cancer‐associated fibroblasts in CRC. (A) UMAP plot of CAFs colored by eight distinct subtypes. (B) Stacked bar plot shows the proportion of each CAF subtype in tumor, border, and normal samples. (C) Sankey plot visualizes the distribution of CAF subtypes across tissue types. (D) Dot plot displaying the expression of top differentially expressed genes across CAF subtypes. (E) Radar chart illustrating average expression of subtype‐specific gene sets via AUCell algorithm. (F) Heatmap of average gene expression across CAF subtypes. (G) Heatmap showing differential pathway activation across CAF subtypes. (H) Heatmap of KEGG pathway enrichment for differentially expressed genes in CAF subtypes. (I) Box plots compare the infiltration levels of eight CAF subtypes in tumor vs. paratumor normal tissues from the TCGA‐COAD dataset.  ^∗∗∗^ indicates *p*  < 0.001; ns indicates no significance.

**Figure:**
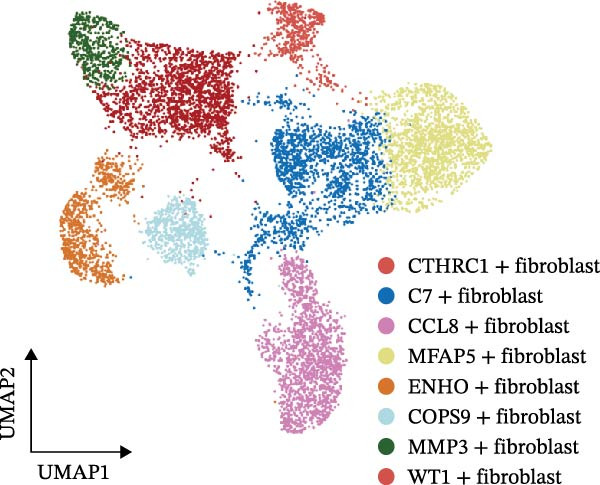
(A)

**Figure:**
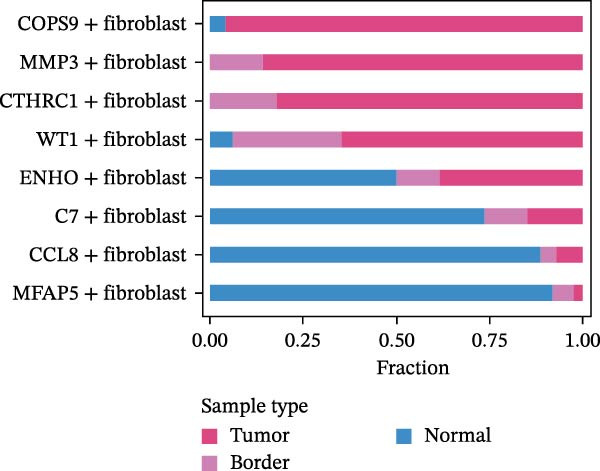
(B)

**Figure:**
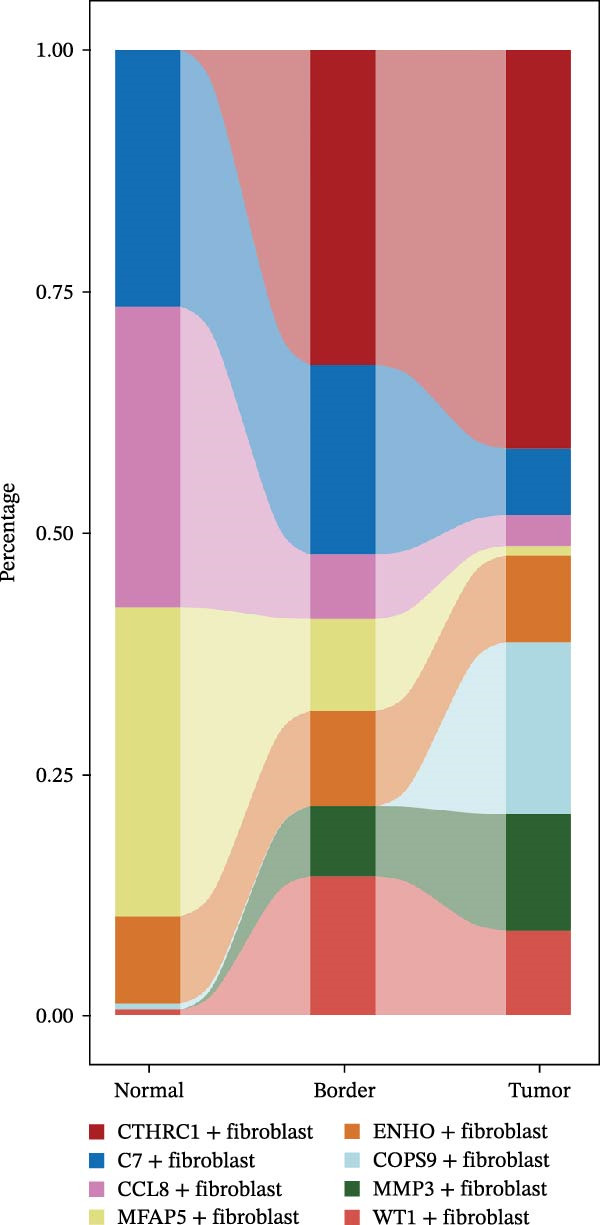
(C)

**Figure:**
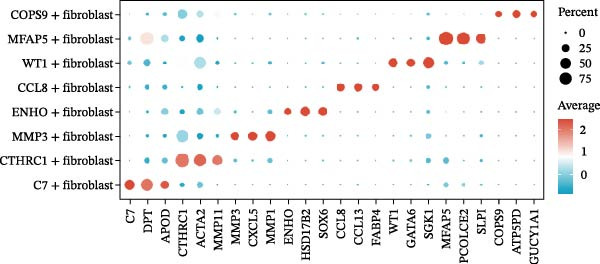
(D)

**Figure:**
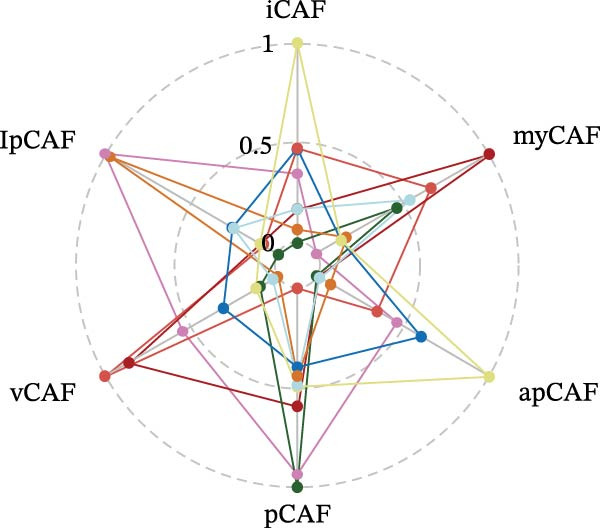
(E)

**Figure:**
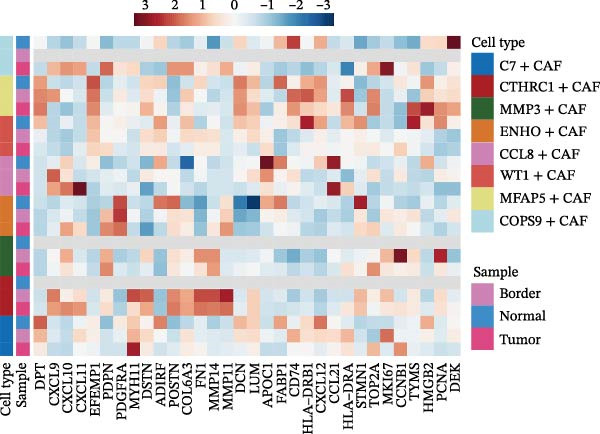
(F)

**Figure:**
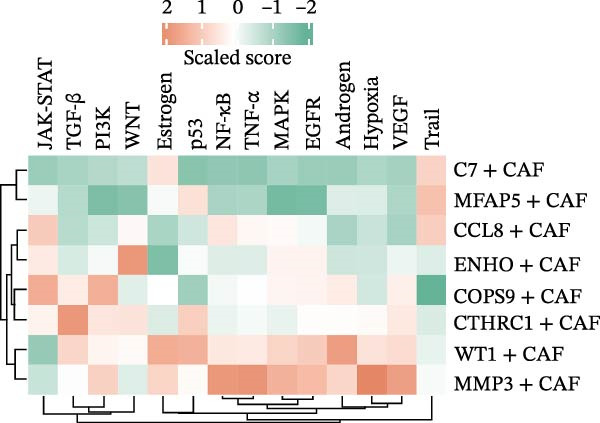
(G)

**Figure:**
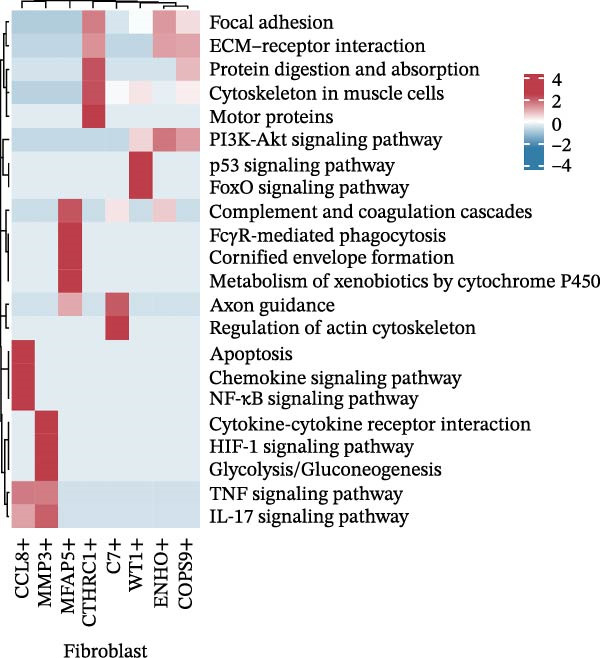
(H)

**Figure:**
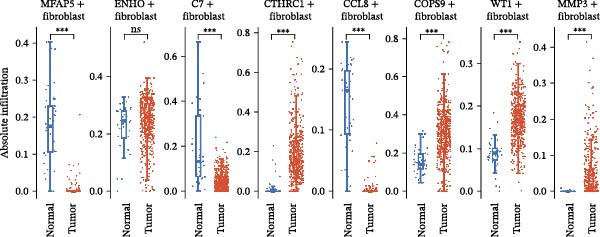
(I)

### 3.3. Pseudotime Analysis of CAF Subtypes in CRC

The inferred developmental trajectory of CAFs was constructed using Monocle2, color‐coded by subtype. The analysis reveals distinct branches corresponding to eight CAF subtypes (Figure 3A). Figure 3B depicts the pseudotime trajectory, with colors ranging from dark blue to light blue indicating increasing pseudotime values. Figure 3C shows the inferred trajectory states, with circles representing the proportion of CAF subtypes within each state. The color coding of each circle corresponds to different CAF subtypes, and the size of the circle indicates the proportion of each subtype within the state. In Figure 3D, a heatmap shows significantly DEGs (q < 1e–3) between two CAF cell fates, MFAP5+ and CCL8+ CAFs. The boxplots on the right display the top five significantly enriched GOBP pathways within each gene cluster. Figure 3E provides a pseudotemporal projection of the transcriptional changes in ECM‐related genes and hypoxia‐related genes, which reveals dynamic shifts in gene expression patterns associated with different CAF subtypes. A heatmap shows the average activity of various transcription factors across different CAF subtypes (Figure 3F), highlighting the transcriptional regulatory landscape within CAF subtypes. Figure 3G presents dot plots of the top six transcription factors specifically activated in each CAF subtype, ranked by their specificity scores. This analysis identifies key transcriptional regulators driving the differentiation and functional heterogeneity of CAF subtypes. Figure 3H shows a UMAP plot of CREB3L1 mRNA expression (left) and violin plots of mRNA gene expression (right). These plots demonstrate the distribution and expression levels of CREB3L1 across different CAF subtypes. Another UMAP plot of CREB3L1 regulon activity scores (left) and violin plots of regulon scores (right) further reveal the regulatory impact of CREB3L1 on downstream target genes within CAF subtypes (Figure 3I). Collectively, these results provide a detailed characterization of the transcriptional dynamics and developmental trajectories of CAF subtypes in CRC, highlighting the heterogeneity and functional diversity of CAFs in the TME.

Figure 3Pseudotime analysis reveals developmental trajectories and transcriptional dynamics of CAF subtypes in CRC. (A) Developmental trajectory of CAFs inferred by Monocle2, colored by subtype. (B) Pseudotime trajectory colored from dark to light blue indicating increasing pseudotime. (C) Trajectory states with pie charts showing proportion of CAF subtypes per state. (D) Heatmap of differentially expressed genes between MFAP5+ and CCL8+ CAF fates, with boxplots of enriched GOBP pathways. (E) Pseudotemporal expression patterns of extracellular matrix and hypoxia‐related genes. (F) Heatmap of transcription factor activity across CAF subtypes. (G) Dot plots of top six transcription factors activated in each CAF subtype. (H) UMAP and violin plots of CREB3L1 mRNA expression across CAF subtypes. (I) UMAP and violin plots of CREB3L1 regulon activity scores.

**Figure:**
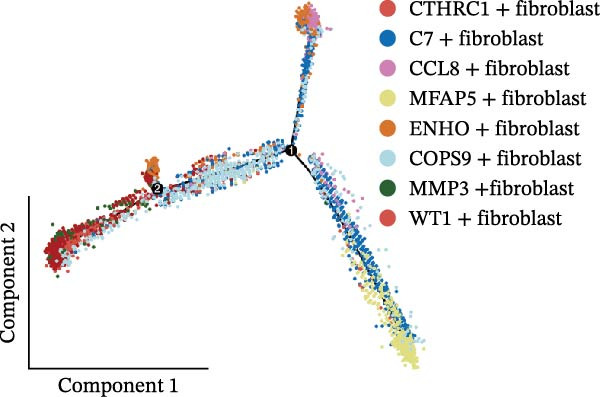
(A)

**Figure:**
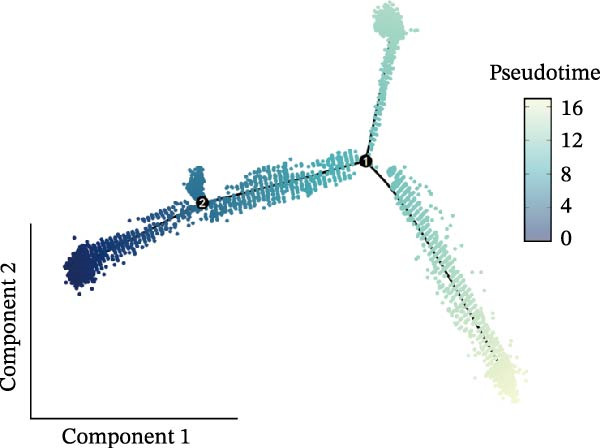
(B)

**Figure:**
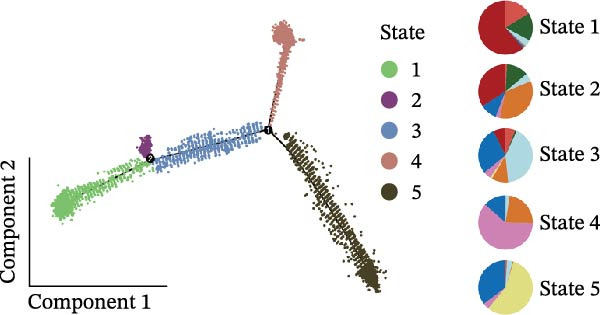
(C)

**Figure:**
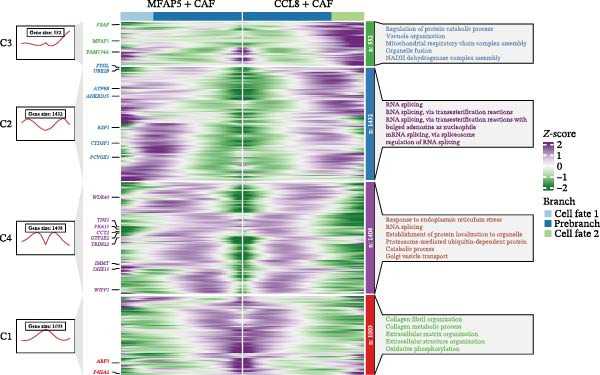
(D)

**Figure:**
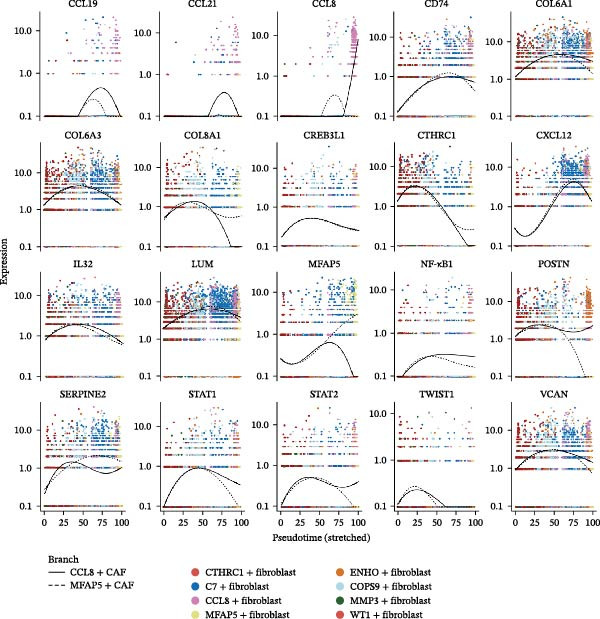
(E)

**Figure:**
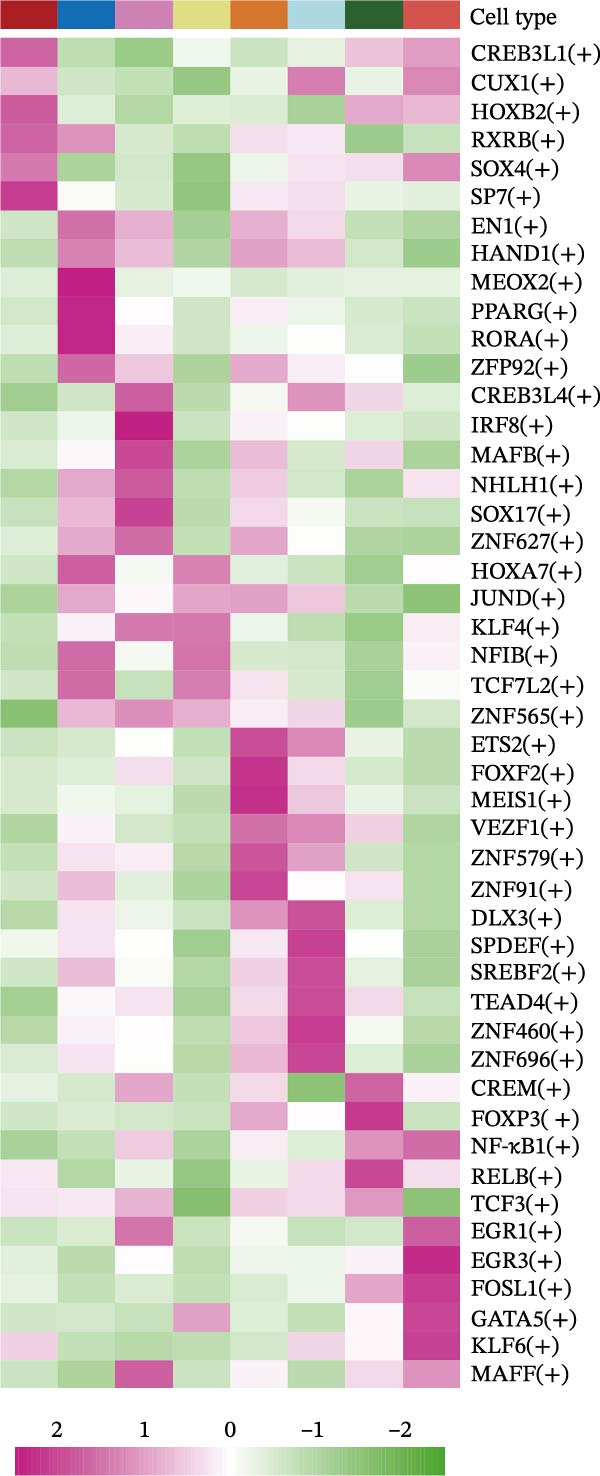
(F)

**Figure:**
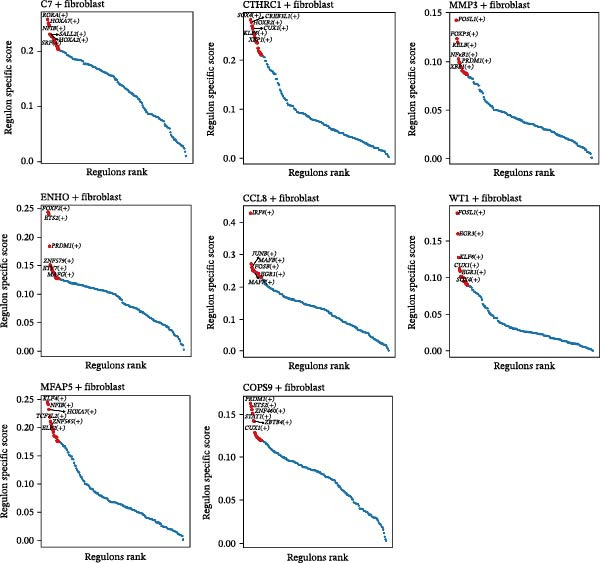
(G)

**Figure:**
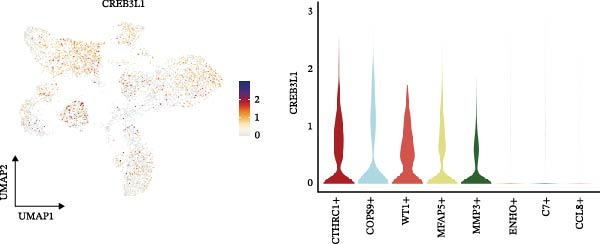
(H)

**Figure:**
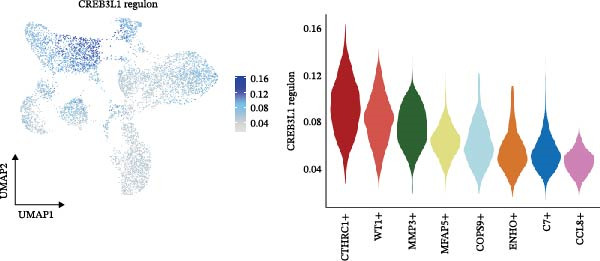
(I)

### 3.4. The Infiltration of CTHRC1+ CAFs Is Significantly Correlated With T Cell Exclusion in CRC

We performed GSEA analysis, and Figure 4A presents the results comparing CTHRC1+ CAFs with other CAF subtypes across various biological pathways, including ECM‐receptor interactions, glycolysis, epithelial–mesenchymal transition (EMT), and focal adhesion. The genes are ranked based on their fold change in expression between the two conditions, highlighting significant pathway enrichments. Figure 4B illustrates the correlation between single‐sample GSEA (ssGSEA) scores of all the eight CAF subtypes and T cell exclusion scores in the TCGA‐COAD cohort, which reveals a significant association between specific CAF subtypes and T cell exclusion. Furthermore, the correlations between the abundance of different CAF subtypes and T cell infiltration abundance in the TCGA‐COAD dataset are depicted in Figure S2. Notably, a positive correlation was not observed between CTHRC1+ CAFs and T cell infiltration. These findings suggest a potential role of CTHRC1+ CAFs in immune evasion. Figure 4C displays spatial clustering maps of three CRC samples, identifying distinct regions enriched in specific CAF subtypes. Figure 4D presents SpatialFeaturePlots of hypoxia, CTHRC1+ fibroblasts, epithelial cells, and T cell features across three CRC sections, providing a spatial overview of the TME and immune cell distribution. Furthermore, spatial expression maps of CTHRC1, COL1A1, EPCAM, and CD3D in three tumor tissues using BayesSpace, highlighting the spatial distribution of these markers within the TME (Figure 4E). Boxplots compare the expression levels of immune checkpoints, including PDCD1, CTLA4, HAVCR2, LAG3, TIGIT, CD276, TNFRSF8, LGALS9, and PDCD1LG2, between high and low infiltration groups of CTHRC1+ CAFs in the TCGA‐COAD cohort; all these genes are significantly elevated in samples featured with high infiltration of CTHRC1+ CAFs (Figure 4F).

Figure 4CTHRC1+ CAFs correlate with T cell exclusion and immune checkpoint upregulation in CRC. (A) GSEA enrichment plots comparing CTHRC1+ CAFs with other subtypes across ECM, glycolysis, EMT, and focal adhesion pathways. (B) Correlation between ssGSEA scores of CAF subtypes and T cell exclusion scores in TCGA‐COAD cohort. (C) Spatial clustering maps of three CRC samples highlighting CAF‐enriched regions. (D) Spatial feature plots of hypoxia, CTHRC1+ fibroblasts, epithelial cells, and T cell markers. (E) Spatial expression maps of CTHRC1, COL1A1, EPCAM, and CD3D in three tumor tissues. (F) Boxplots comparing immune checkpoint gene expression between high and low CTHRC1+ CAF infiltration groups.  ^∗^ indicates *p* < 0.05;  ^∗∗∗^ indicates *p* < 0.001.

**Figure:**
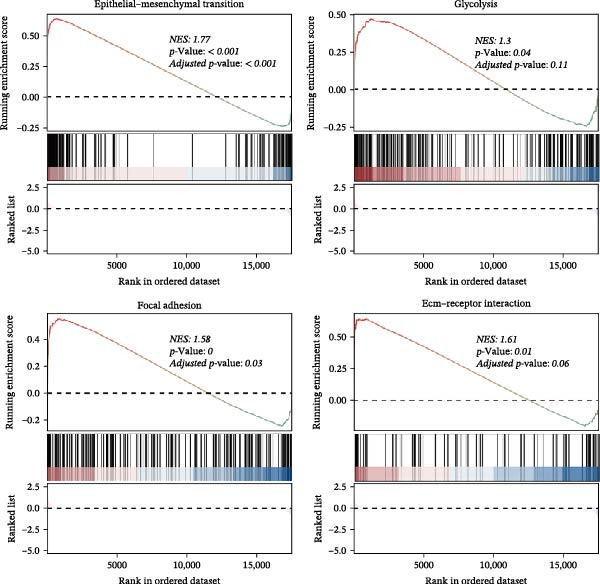
(A)

**Figure:**
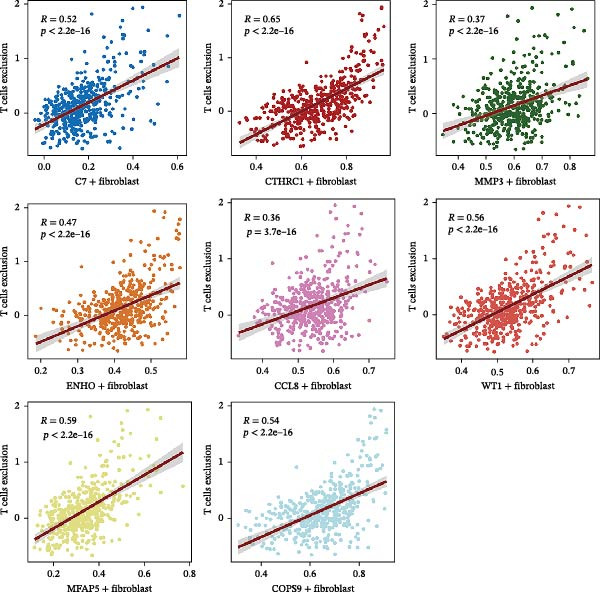
(B)

**Figure:**
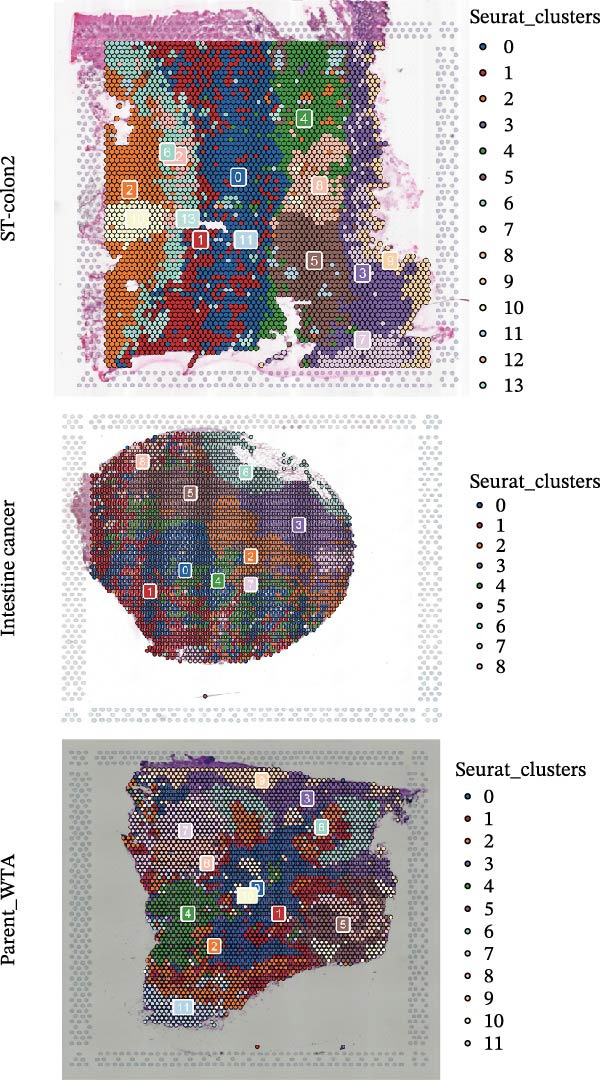
(C)

**Figure:**
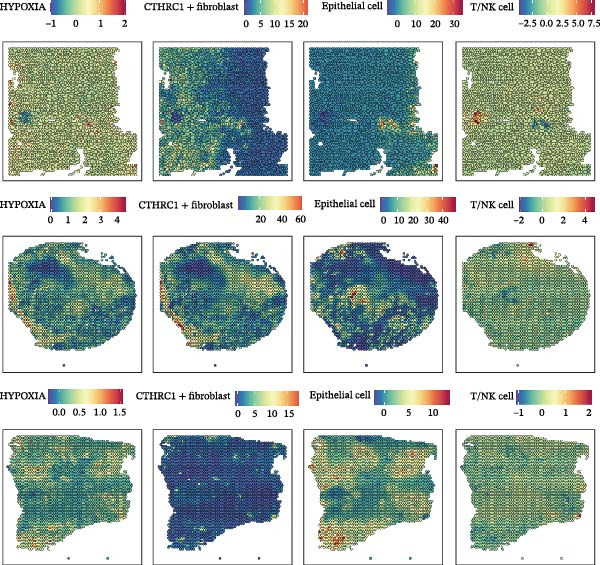
(D)

**Figure:**
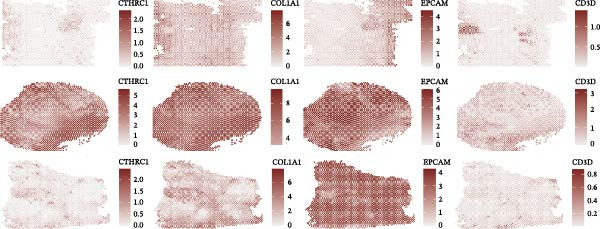
(E)

**Figure:**
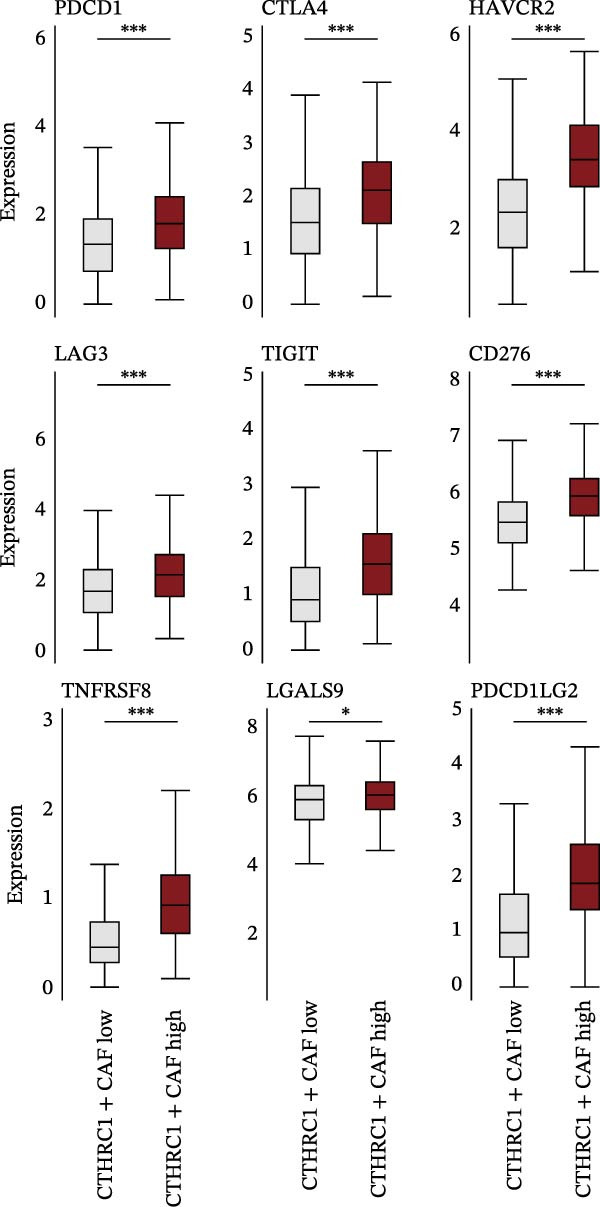
(F)

### 3.5. Heterogeneity and Plasticity of Epithelial Cells in CRC

Figure 5A presents a heatmap of the Jaccard similarity index based on the GeneNMF decomposition algorithm, illustrating the robust NMF programs among epithelial cells. The programs are systematically classified into several metaprogram families (indicated by black boxes), with biological features of GOBP enrichment annotated on the right side of the heatmap. This reveals the intrinsic modular structure of transcriptional programs within epithelial cells. Violin plots show the gene set scores for MetaPrograms (MP1 to MP9) calculated using the AddModuleScore function, quantifying the expression levels of these programs across various epithelial cell types (Figure 5B). Figure 5C displays FeaturePlot UMAP maps of the gene set scores for MP1 to MP9, providing a visual representation of the distribution of these programs within the epithelial cell population. A UMAP map depicts epithelial cells colored by different subtypes, demonstrating cellular heterogeneity of Malig‐Epi cells (Figure 5D). Figure 5E shows a UMAP map of epithelial cells colored by tissue type, highlighting the distribution of epithelial cells across different tissue samples. Figure 5F illustrates a heatmap of the *z*‐score normalized expression levels of the top eight marker genes in each epithelial subtype, providing insights into the molecular characteristics of these subtypes. Bar charts show GOBP enrichment analysis of the top 100 marker genes in each epithelial subgroup, further elucidating the biological functions associated with these subgroups (Figure 5G). Figure 5H displays scatter plots showing the infiltration proportion of epithelial subtypes in tissue types (normal, boundary, and tumor), with *p*‐values calculated using *t*‐tests for intergroup comparisons. Figure 5I presents a UMAP projection map of epithelial subgroup cells along the trajectory inferred by Monocle3, with cells colored according to pseudotime, and ridgeline plots illustrate the dynamic changes of each epithelial cell subtype along pseudotime (Figure 5J). Furthermore, CNV scores were estimated using the inferCNV algorithm, and a heatmap displays the results for epithelial cells, using immune cells as a reference (Figure S3A). A violin plot shows CNV scores for different cell types (Figure S3B). Compared to other cells, the CNV score of epithelial cells is the highest (*p* < 2.2e–16). Figure 5K presents a UMAP map of epithelial cells colored by CNV scores, and the level of CNVs in each sample source was shown in Figure S3C. In addition, violin plots display CNV scores for epithelial cell subsets (Figure 5L).

Figure 5Heterogeneity and plasticity of epithelial cells in CRC. (A) Heatmap of Jaccard similarity index showing robust NMF programs among epithelial cells. (B) Violin plots of gene set scores for MetaPrograms MP1–MP9 across epithelial cell types. (C) FeaturePlot UMAP maps of MP1–MP9 gene set scores. (D) UMAP map of epithelial cells colored by subtype. (E) UMAP map of epithelial cells colored by tissue type. (F) Heatmap of *z*‐score normalized expression of top eight marker genes per epithelial subtype. (G) Bar charts of GOBP enrichment for differentially expressed genes per epithelial subgroup. (H) Scatter plots of epithelial subtype infiltration proportion across tissue types. (I) UMAP trajectory of epithelial cells inferred by Monocle3, colored by pseudotime. (J) Ridgeline plots of epithelial subtype dynamics along pseudotime. (K) UMAP map of epithelial cells colored by CNV scores. (L) Violin plots of CNV scores across epithelial subgroups.

**Figure:**
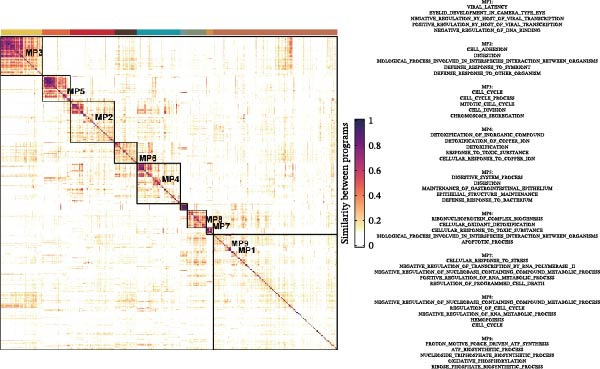
(A)

**Figure:**
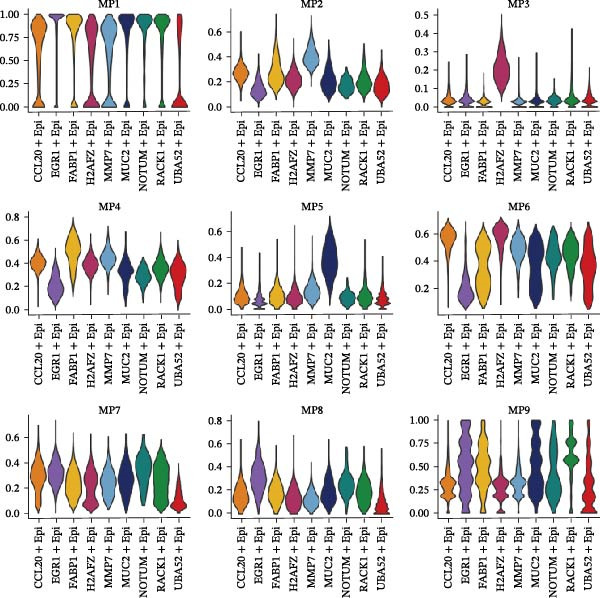
(B)

**Figure:**
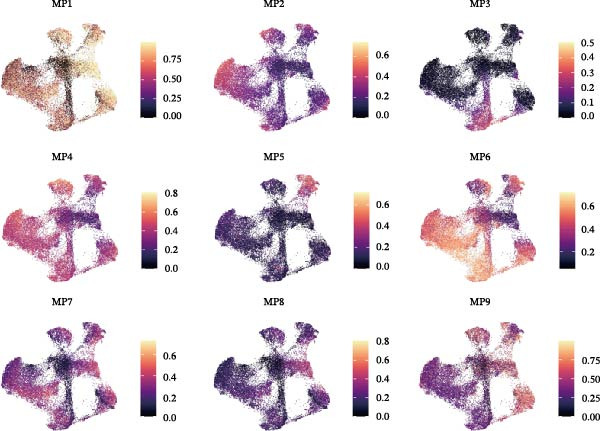
(C)

**Figure:**
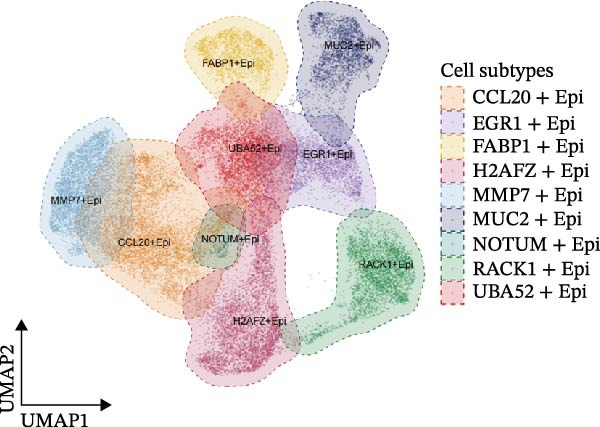
(D)

**Figure:**
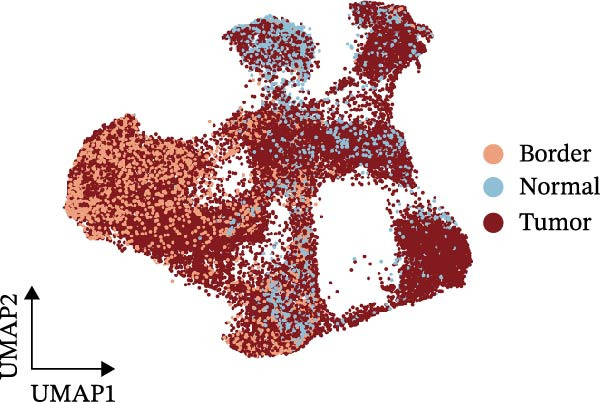
(E)

**Figure:**
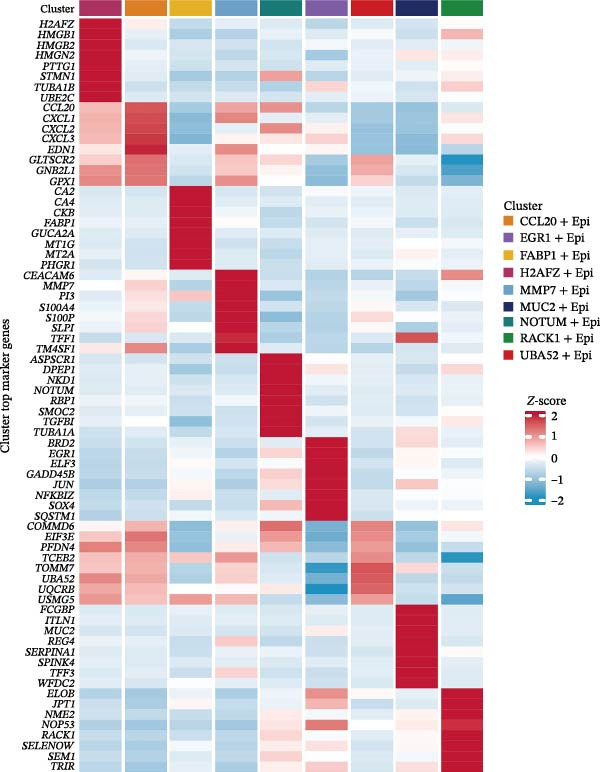
(F)

**Figure:**
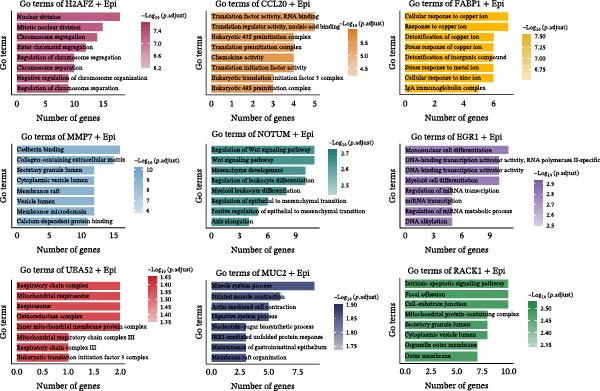
(G)

**Figure:**
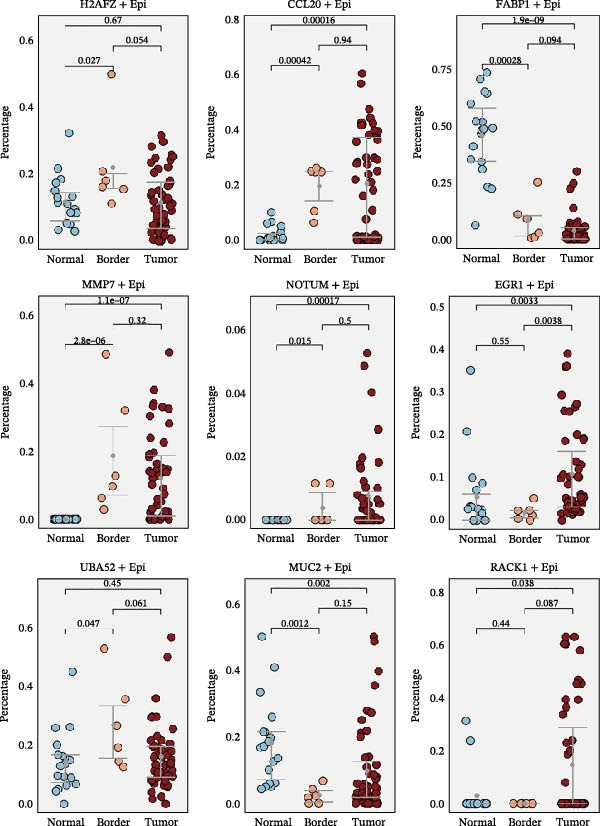
(H)

**Figure:**
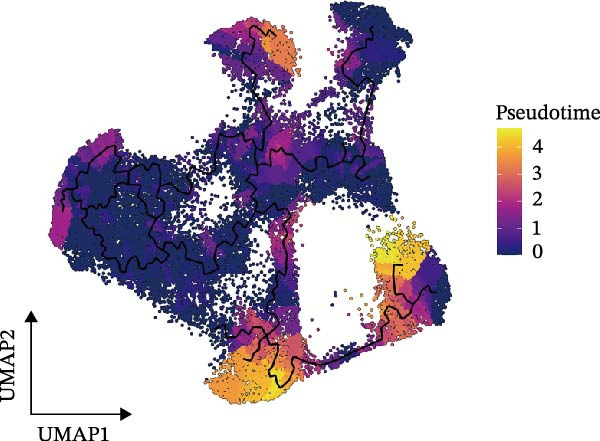
(I)

**Figure:**
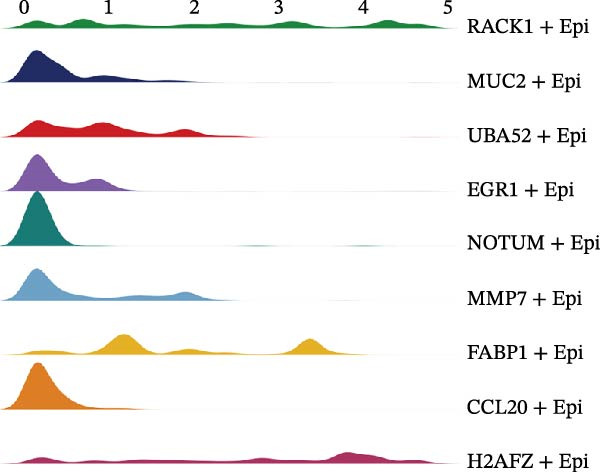
(J)

**Figure:**
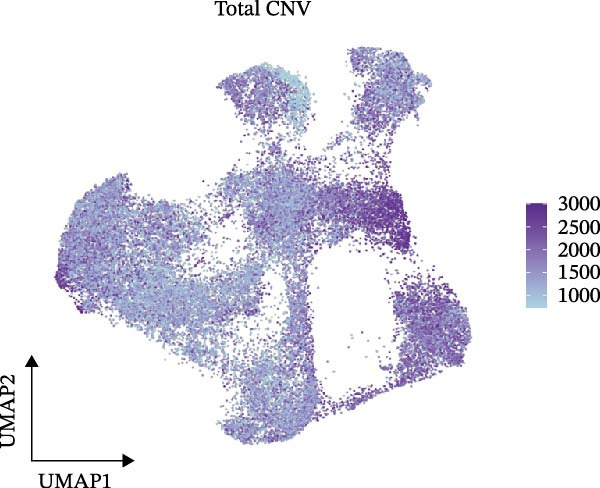
(K)

**Figure:**
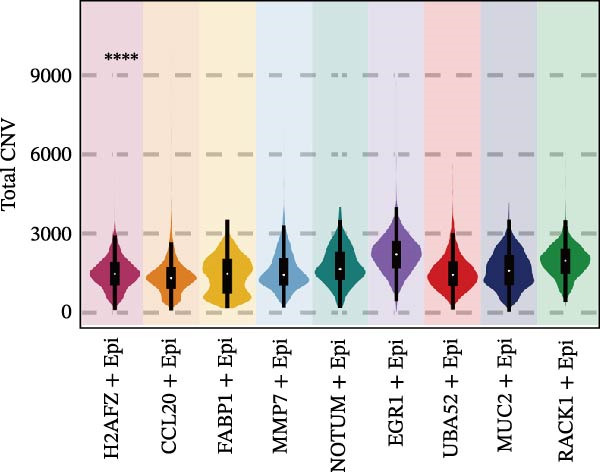
(L)

### 3.6. CTHRC1+ CAFs Communicate With MMP7+ Malig‐Epi Cells via the THBS2‐SDC4 Signaling Pathway

A circular plot depicts the cell communication network, comparing the number and strength of interactions between fibroblasts and epithelial cell subtypes in malignant versus nonmalignant samples, which highlights the complex intercellular communication patterns in the TME (Figure 6A). Figure 6B presents bubble charts that detail the communication patterns, with the upper chart showing the reception modes of target cells and the lower chart displaying the emission modes of secretory cells. Figure 6C compares the intensity of signaling pathways between tumor and adjacent normal tissues, alongside the number and strength of cellular interactions between the two groups. Figure 6D shows a communication heatmap indicating the most intimate interactions between CTHRC1+ CAFs and MMP7+ Malig‐Epi cells, suggesting a significant role in tumor progression. Circular plots display the number of interactions of CTHRC1+ CAFs with other cell types (top) and MMP7+ Malig‐Epi cells with other cell types (bottom), emphasizing their central roles in cellular communication (Figure 6E). Figure 6F presents a two‐dimensional scatter plot analyzing the signaling roles of CAF subtypes and epithelial subtypes, with MMP7+ Malig‐Epi cells identified as the most active signal emitters and CTHRC1+ CAFs as the most active signal receivers. Figure 6G illustrates the interaction network of all cell types within the THBS signaling pathway, highlighting the interconnectedness of various cell types in this pathway. The THBS2‐SDC4 signaling network was detected in various cell types, especially in the CTHRC1+ CAFs, indicating the pivotal roles of certain cells in this pathway (Figure 6H). Figure 6I shows the centrality scores of the THBS signaling network, further emphasizing the key players in this pathway. Figure 6J displays the expression levels of various ligand–receptor proteins across different cell types within the THBS signaling pathway. Figure 6K features a chord diagram depicting the interactions between CTHRC1+ CAFs and MMP7+ Malig‐Epi cells, visually representing the intensity of their communication. Figure 6L illustrates the interactions among all cells within the THBS2‐SDC4 pathway, which provides a comprehensive view of the cellular crosstalk mediated by this pathway.

Figure 6CTHRC1+ CAFs communicate with MMP7+ malignant epithelial cells via THBS2‐SDC4 signaling. (A) Circular plot comparing fibroblast–epithelial interactions in malignant vs. nonmalignant samples. (B) Bubble charts detailing communication patterns: reception (top) and emission (bottom). (C) Comparison of signaling intensity and interaction strength between tumor and normal tissues. (D) Communication heatmap highlighting interactions between CTHRC1+ CAFs and MMP7+ malignant epithelial cells. (E) Circular plots of interaction numbers for CTHRC1+ CAFs (top) and MMP7+ malignant epithelial cells (bottom). (F) Scatter plot of signaling roles, identifying MMP7+ cells as top emitters and CTHRC1+ CAFs as top receivers. (G) Interaction network of all cell types within THBS signaling pathway. (H) THBS2‐SDC4 signaling network across cell types. (I) Centrality scores of THBS signaling network. (J) Expression levels of ligand–receptor pairs in THBS signaling pathway. (K) Chord diagram depicts interactions between CTHRC1+ CAFs and MMP7+ malignant epithelial cells. (L) Interaction network within THBS2‐SDC4 pathway.

**Figure:**
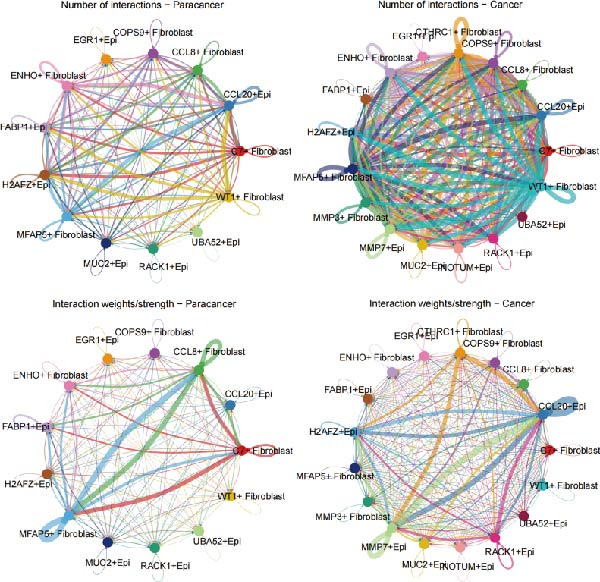
(A)

**Figure:**
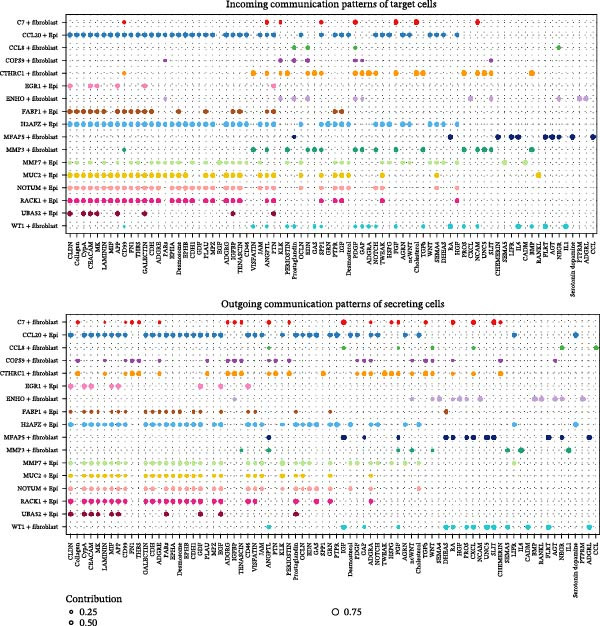
(B)

**Figure:**
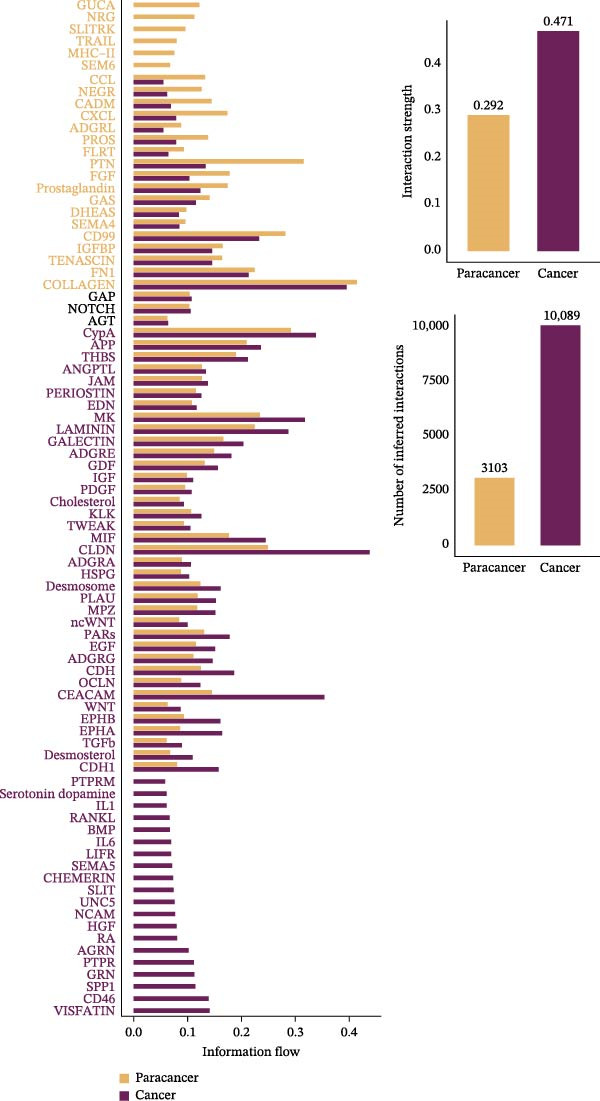
(C)

**Figure:**
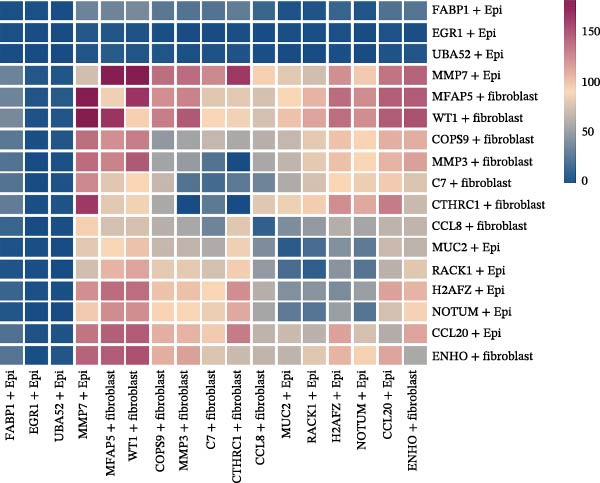
(D)

**Figure:**
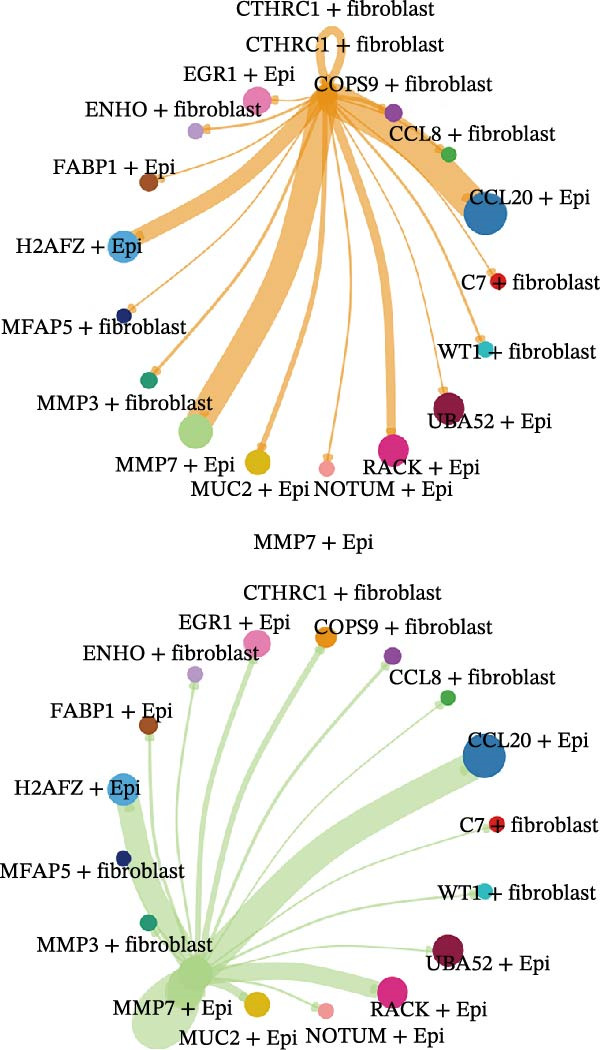
(E)

**Figure:**
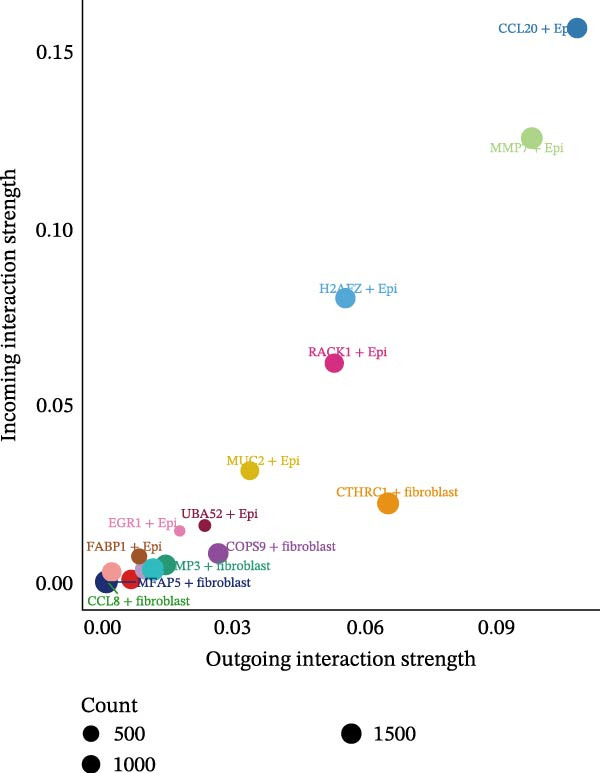
(F)

**Figure:**
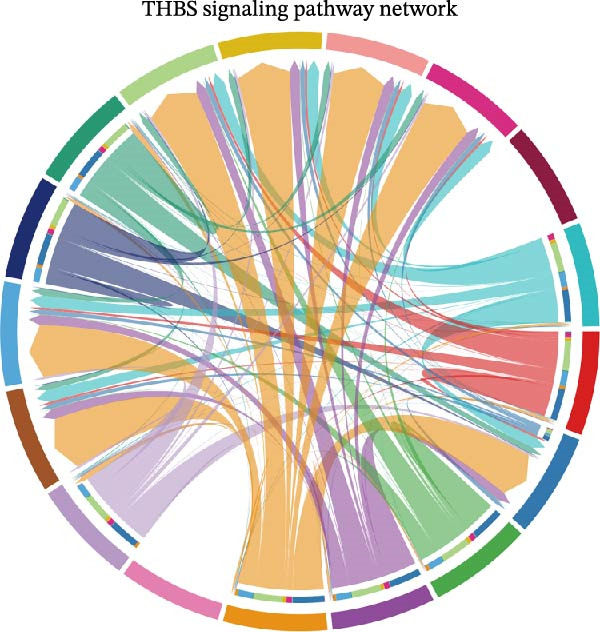
(G)

**Figure:**
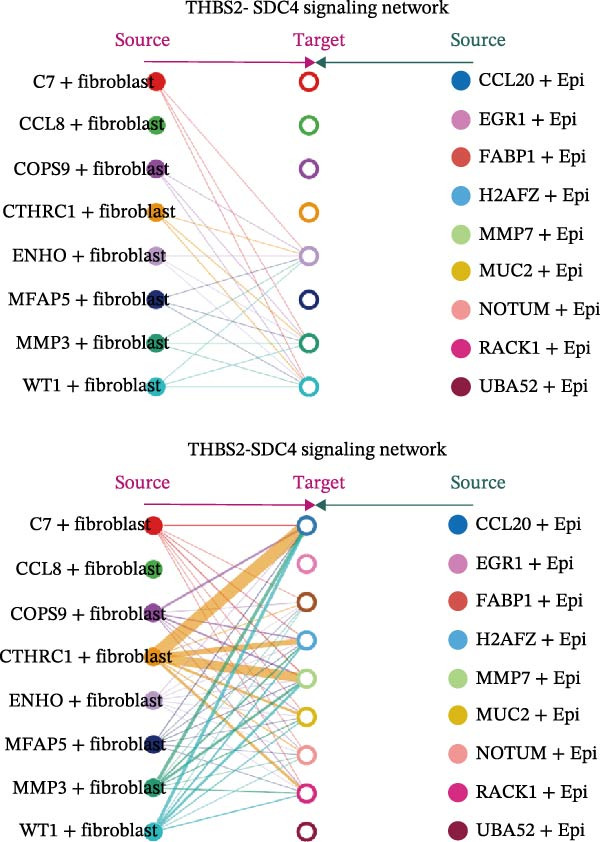
(H)

**Figure:**
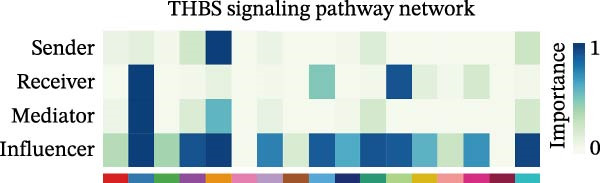
(I)

**Figure:**
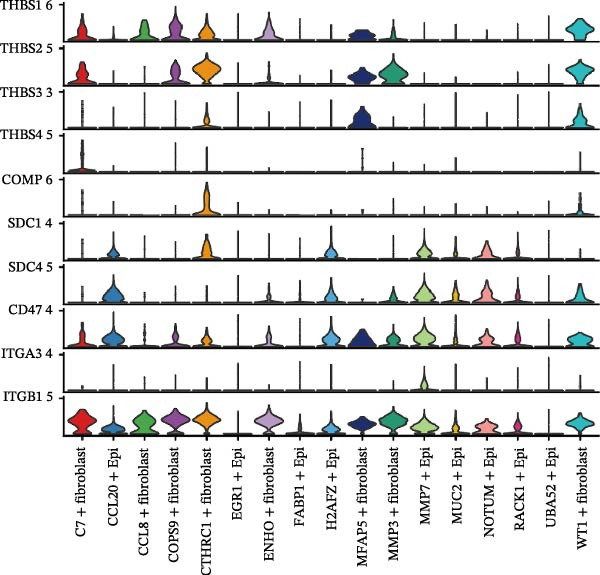
(J)

**Figure:**
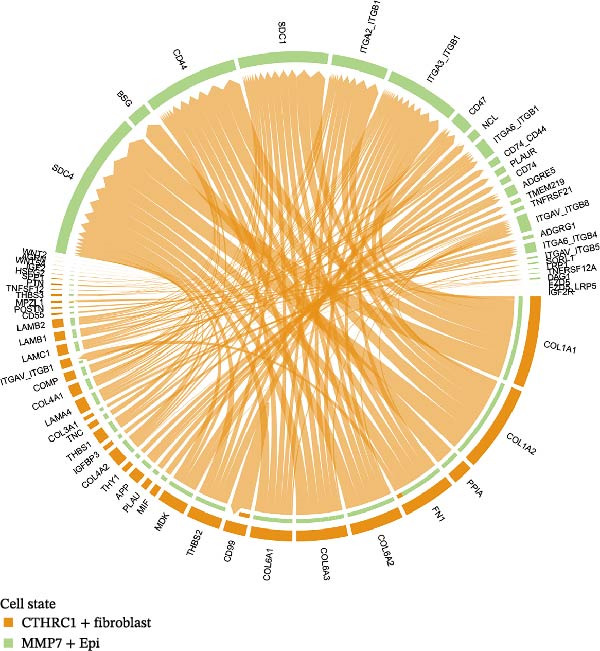
(K)

**Figure:**
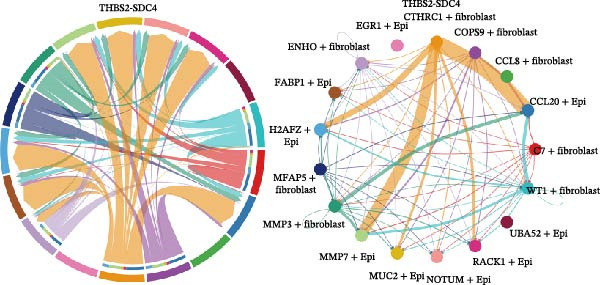
(L)

### 3.7. Association of CTHRC1+ CAFs and MMP7+ Malig‐Epi Cell Infiltration With Poor Clinical Outcomes in CRC Patients

Figure 7A presents survival analyses using Kaplan–Meier curves for patients in the TCGA‐COAD cohort, comparing low and high infiltration levels of CTHRC1+ CAFs alone (left), MMP7+ Malig‐Epi alone (middle), and the combination of both (right). The results indicate that higher infiltration levels of CTHRC1+ CAFs and MMP7+ Malig‐Epi are associated with poorer prognosis. Figure 7B shows similar survival analyses in the anti‐PD‐L1 immunotherapy cohort (IMvigor210), again using Kaplan–Meier curves for low and high infiltration levels of CTHRC1+ CAFs (left), MMP7+ Malig‐Epi (middle), and the combination of both (right). These analyses further confirm that high infiltration levels of CTHRC1+ CAFs and MMP7+ Malig‐Epi cells suggest a worse prognosis. A stacked plot reveals that patients with concurrent high infiltration of CTHRC1+ CAFs and MMP7+ Malig‐Epi cells have a higher proportion of progressive disease (PD) or stable disease (SD), suggesting a potential resistance to treatment (Figure 7C). A heatmap shows the Jaccard similarity coefficients of gene programs identified by SpaGene across various spatially resolved samples, calculated based on the overlap of top‐ranked genes, which highlights the modular structure of transcriptional programs within the TME (Figure 7D). Spatial feature plots display specific gene patterns, CTHRC1+ CAFs, and MMP7+ Malig‐Epi characteristics in spatially resolved tumor tissues from four CRC patients (from left to right; Figure 7E). The scatter plots demonstrate the Spearman correlation coefficients between CTHRC1+ CAFs and MMP7+ Malig‐Epi feature scores, spatially confirming a significant positive correlation between the two.

Figure 7High infiltration of CTHRC1+ CAFs and MMP7+ malignant epithelial cells correlates with poor clinical outcomes in CRC. (A) Kaplan–Meier survival curves for TCGA‐COAD cohort stratified by CTHRC1+ CAFs (left), MMP7+ malignant epithelial cells (middle), and combined infiltration (right). (B) Survival analysis in IMvigor210 immunotherapy cohort for the same cell types. (C) Stacked plot shows treatment response (PD/SD vs. CR/PR) in patients with high dual infiltration, indicating patients with distinct infiltrating patterns of CTHRC1+ CAFs and MMP7+ malignant epithelial cells exhibited different responses to immunotherapy. (D) Heatmap of Jaccard similarity coefficients for spatial gene programs across CRC samples. (E) Spatial feature plots of CTHRC1+ CAFs and MMP7+ malignant epithelial characteristics in four CRC patients; scatter plots show spatial correlation between CTHRC1+ CAFs and MMP7+ malignant epithelial feature scores.

**Figure:**
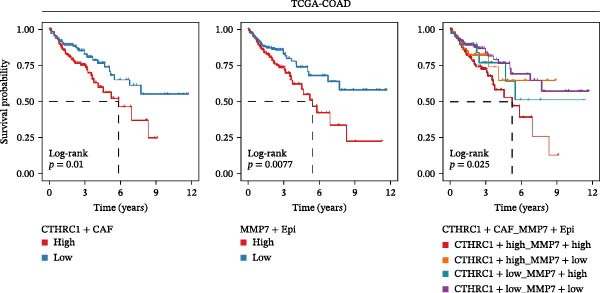
(A)

**Figure:**
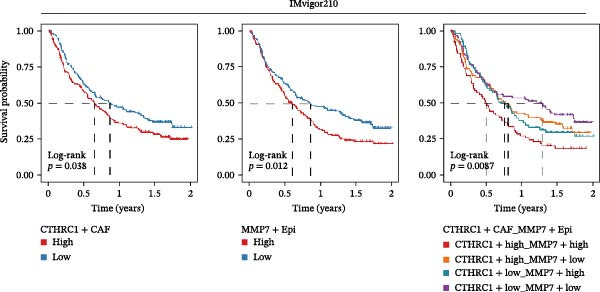
(B)

**Figure:**
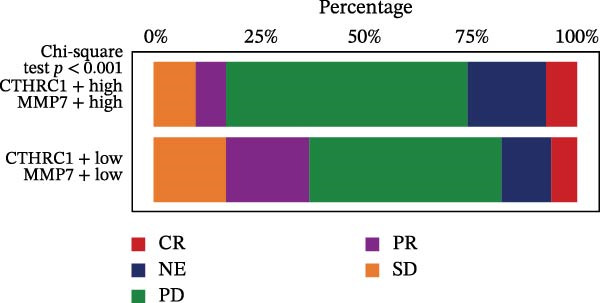
(C)

**Figure:**
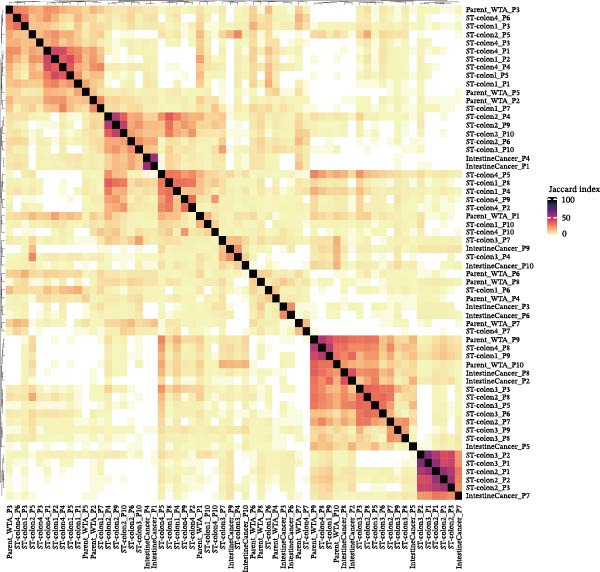
(D)

**Figure:**
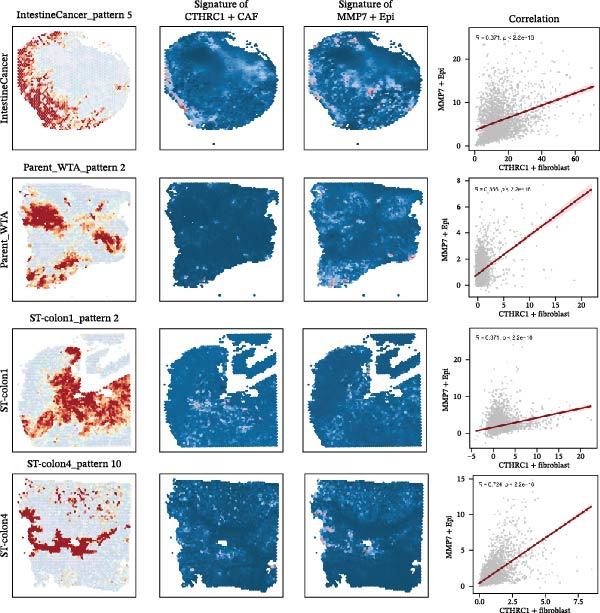
(E)

## 4. Discussion

CRC presents as a highly heterogeneous disease, not only at the genomic level of malignant cells but also in the complex composition and dynamic interactions within its TME [27, 28]. This profound heterogeneity underpins the variable clinical trajectories and differential treatment responses observed among patients, posing a major challenge to the efficacy of standardized therapies [29, 30]. Therefore, deconstructing the cellular and molecular architecture of the TME, with a particular focus on key orchestrators like CAFs, is not merely an academic exercise but a critical prerequisite for advancing precision oncology. A deeper understanding of CAF plasticity, their functional subsets, and their communication networks is essential to identify predictive biomarkers and develop targeted strategies that can disrupt protumorigenic niches and overcome therapeutic resistance, ultimately paving the way for more effective, individualized patient care.

In this study, we have leveraged high‐resolution single‐cell and spatial transcriptomic technologies to systematically dissect the heterogeneity, plasticity, and functional roles of CAFs within the CRC microenvironment. Our integrated analysis reveals a complex ecosystem wherein distinct CAF subtypes exhibit unique transcriptional profiles, developmental trajectories, and specific interactions with other cellular components, notably Malig‐Epi cells. These findings not only deepen our understanding of CAF biology in CRC but also unveil potential mechanistic links to tumor progression, immune evasion, and therapeutic resistance.

A central finding of our work is the identification and characterization of eight functionally distinct CAF subtypes in CRC, including CTHRC1+, C7+, CCL8+, MFAP5+, ENHO+, COPS9+, MMP3+, and WT1+ fibroblasts. The presence of these subtypes, each with a specific gene expression signature and pathway activation profile, underscores the remarkable plasticity and contextual adaptation of CAFs within the TME. Notably, the distribution of these subtypes varied significantly between tumor, border, and normal tissues, suggesting that local environmental cues—such as hypoxia, metabolic stress, or immune pressure—drive CAF differentiation into specialized functional states. For instance, our pseudotime trajectory analysis indicates branching points that may represent fate decisions, guiding CAFs towards profibrotic, immunomodulatory, or matrix‐remodeling phenotypes. This developmental model provides a framework for understanding how CAF heterogeneity arises and evolves during CRC progression.

Among the identified subtypes, CTHRC1+ CAFs emerged as a particularly consequential population. Our data strongly associate this subtype with features of an immunosuppressive and protumorigenic microenvironment. GSEA revealed significant enrichment of pathways related to ECM organization, EMT, and glycolysis in CTHRC1+ CAFs, aligning with their potential role in constructing a dense, fibrotic stroma and promoting tumor invasion. Crucially, we found a significant positive correlation between CTHRC1+ CAF infiltration and T cell exclusion scores in the TCGA cohort. Spatial transcriptomics further visualized the colocalization of CTHRC1+ CAF‐rich regions with areas of hypoxia and depletion of T cells, providing spatial evidence for their role in creating an immune‐privileged niche. Moreover, samples with high CTHRC1+ CAF infiltration exhibited elevated expression of multiple immune checkpoint molecules (e.g., PDCD1, CTLA4, and LAG3), suggesting that this CAF subset may contribute to adaptive immune resistance, potentially rendering tumors less responsive to ICB therapy.

Equally important is our discovery of a specific and robust communication axis between CTHRC1+ CAFs and MMP7+ Malig‐Epi cells, mediated primarily by the THBS2‐SDC4 ligand–receptor pair. Cell–cell interaction analysis positioned CTHRC1+ CAFs as dominant signal receivers and MMP7+ epithelial cells as prominent signal emitters within this pathway. The THBS family is known to be involved in cell adhesion, migration, and activation of latent TGF‐β, a master regulator of stromal activation and immune suppression. The specific THBS2‐SDC4 interaction uncovered here provides a mechanistic hypothesis for how CAFs and cancer cells cooperatively reinforce a malignant microenvironment. This paracrine signaling loop could enhance ECM deposition, epithelial plasticity, and ultimately, tumor growth and metastasis.

The clinical relevance of our findings is underscored by survival analyses. High infiltration levels of either CTHRC1+ CAFs or MMP7+ Malig‐Epi cells were independently associated with poorer overall survival in the TCGA‐COAD cohort. Strikingly, patients with concurrent high infiltration of both cell types exhibited the worst prognosis. This synergistic effect was corroborated in an independent immunotherapy cohort (IMvigor210), where the same combination was linked to a higher rate of progressive or SD following anti‐PD‐L1 therapy. These results posit the combined signature of CTHRC1+ CAFs and MMP7+ epithelial cells as a promising, biologically grounded biomarker for risk stratification and prediction of immunotherapy resistance in CRC. The spatial colocalization of CTHRC1+ CAFs and MMP7+ epithelial cells, confirmed by our spatial transcriptomic data, moves beyond correlation to suggest direct and localized crosstalk within tumor regions.

The THBS2‐SDC4 axis offers a promising therapeutic target for disrupting protumorigenic crosstalk in high‐risk CRC. While SDC4 is ubiquitously expressed, its functional role in mediating CAF–epithelial communication provides a context‐specific targeting opportunity. Preclinical studies demonstrate that SDC4 silencing inhibits tumor progression, and bufalin‐mediated SDC4 targeting suppresses cancer cell proliferation [31]. High SDC4 expression was observed in CRC and predicts poor prognosis [32]. For immunotherapy‐resistant patients, blocking the THBS2‐SDC4 loop may reprogram the immunosuppressive niche and overcome T cell exclusion. Future strategies include THBS2‐neutralizing antibodies, tumor‐specific SDC4 inhibitors, or dual‐targeting approaches combining CAF‐directed agents with ICB.

We admit that our study has limitations. First, the IMvigor210 dataset represents a urothelial carcinoma cohort treated with anti‐PD‐L1 therapy, rather than a CRC‐specific immunotherapy cohort. While this analysis provides supportive evidence for the association between CTHRC1+ CAF/MMP7+ epithelial infiltration and immunotherapy resistance across cancer types, the findings should be interpreted with caution. This study is primarily based on transcriptional data; functional validation of the identified CAF subtypes and the THBS2‐SDC4 axis using in vitro and in vivo models is necessary to establish causality. Future experimental work is essential to move from correlation to mechanism. For instance, in vitro co‐culture systems of sorted or induced CTHRC1+ CAFs with MMP7+ CRC organoids or cell lines would allow direct testing of the THBS2‐SDC4 mediated crosstalk, enabling assessment of its impact on epithelial cell proliferation, invasion, and drug sensitivity. Furthermore, employing patient‐derived organoids (PDOs) incorporating autologous CAFs would provide a more physiologically relevant platform to dissect the functional consequences of this interaction within a near‐native TME context and to screen for potential therapeutic interventions aimed at disrupting it. Therefore, future studies should focus on the crosstalk between these cell subtypes to elucidate the detailed signaling pathways and molecular mechanisms that drive their interactions, ultimately translating these insights into targeted therapeutic strategies for CRC patients.

## 5. Conclusions

In conclusion, our multiomics integrative study provides a comprehensive atlas of CAF heterogeneity in CRC and delineates a pivotal cellular crosstalk network within the TME. We propose that the CTHRC1+ CAF subtype acts as a key orchestrator of an immune‐suppressive and fibrotic niche, and its communication with MMP7+ Malig‐Epi cells via THBS2‐SDC4 signaling is a potential driver of tumor progression and therapy resistance. These insights shift the perspective of the TME from a static backdrop to a dynamic interplay of specialized cellular communities. Future therapeutic strategies for CRC could be significantly improved by simultaneously targeting Malig‐Epi cells and their supportive CAF partners. Specifically, disrupting the THBS2‐SDC4 axis or modulating the differentiation of CTHRC1+ CAFs may offer novel avenues to reprogram the TME, overcome immune evasion, and enhance the efficacy of existing therapies, ultimately improving outcomes for patients with CRC.

## Author Contributions

Conceptualization: Sisi Wu. Data curation: Yang Yang and Song Huang. Formal analysis: Yang Yang and Yingjian Wang. Funding acquisition: Song Huang and Hanyu Zhou. Supervision: Sisi Wu. Writing – review and editing: Yang Yang and Sisi Wu.

## Funding

This work was supported by the Traditional Chinese Medicine and Ethnic Medicine Scientific and Technological Research Project, Guizhou Provincial Administration of Traditional Chinese Medicine (Grant QZYY‐2025‐117 to Song Huang), the Postdoctoral Program Project of The Third Affiliated Hospital of Soochow University (Grant KY20252302 to Hanyu Zhou), Changzhou “Longcheng Qiangyi” Talent Program (Grant KY20242211 to Hanyu Zhou), Study on the Synergistic Effects and Mechanisms of Combined Treatment with Apatinib and IL‐33‐CEA‐CAR‐T Cells for Gastric Cancer (Grant KY20252460 to Hanyu Zhou), and Changzhou Applied Basic Research Program (pre‐funding) (Grant CJ20251029 to Hanyu Zhou).

## Disclosure

All authors have read and approved the final manuscript.

## Ethics Statement

This study is based on published or public datasets and does not require ethical approval and consent.

## Conflicts of Interest

The authors declare no conflicts of interest.

## Supporting Information

Additional supporting information can be found online in the Supporting Information section.

## Supporting information


**Supporting Information** Figure S1. The distribution characteristics and gene expression profiles of different subtypes of CAFs in CRC samples. (A) UMAP plot shows that CAFs are clustered into eight distinct subtypes. (B) The distribution of these CAFs in normal, border, and tumor samples. (C) The radar chart shows the distribution of cell cycle states among different CAF subtypes. (D) The UMAP plot shows the distribution of the phenotypic scores of six established subtypes among all CAFs in our study; the color scale represents the gradient of the scores. (E) The heatmap shows the average expression levels of lipoxygenase (LOX)‐ and matrix metalloproteinase (MMP)‐related genes in different CAF subtypes. (F) The heatmap shows the average expression levels of collagen (COL)‐related genes in different CAF subtypes. Figure S2. The correlations between the abundance of different CAF subtypes and T cell infiltration abundance in the TCGA‐COAD dataset. Figure S3. CNV scores were estimated using inferCNV algorithm and were compared among different cell types. (A) The heatmap of inferCNV results for epithelial cells, using immune cells as a reference. (B) The violin plot of CNV scores for different cell types; compared to other cells, the CNV score of epithelial cells is the highest. (C) The violin plot of CNV scores for epithelial cells from different sources (normal, border, and tumor).

## Data Availability

The data that support the findings of this study are available from the corresponding author upon reasonable request.
