# Supplementary material for: Integrative Single‐Cell and Spatial Transcriptomics Reveals the Crosstalk of CTHRC1+ CAF and MMP7+ Epithelial Axis as a Potential Therapeutic Target and Predicts Poor Clinical Outcomes in Colorectal Cancer
Source: Mediators Inflamm. 2026 Apr 8;2026:9314553. doi: 10.1155/mi/9314553 (PMC13061587; doi:10.1155/mi/9314553)
Supplement: Supplementary file 1 — Supporting Information Figure S1. The distribution characteristics and gene expression profiles of different subtypes of CAFs in CRC samples. (A) UMAP plot shows that CAFs are clustered into eight distinct subtypes. (B) The distribution of these CAFs in normal, border, and tumor samples. (C) The radar chart shows the distribution of cell cycle states among different CAF subtypes. (D) The UMAP plot shows the distribution of the phenotypic scores of six established subtypes among all CAFs in our study; the color scale represents the gradient of the scores. (E) The heatmap shows the average expression levels of lipoxygenase (LOX)‐ and matrix metalloproteinase (MMP)‐related genes in different CAF subtypes. (F) The heatmap shows the average expression levels of collagen (COL)‐related genes in different CAF subtypes. Figure S2. The correlations between the abundance of different CAF subtypes and T cell infiltration abundance in the TCGA‐COAD dataset. Figure S3. CNV scores were estimated using inferCNV algorithm and were compared among different cell types. (A) The heatmap of inferCNV results for epithelial cells, using immune cells as a reference. (B) The violin plot of CNV scores for different cell types; compared to other cells, the CNV score of epithelial cells is the highest. (C) The violin plot of CNV scores for epithelial cells from different sources (normal, border, and tumor). [file MI-2026-9314553-s001.docx]

**Supplementary figures and legends**


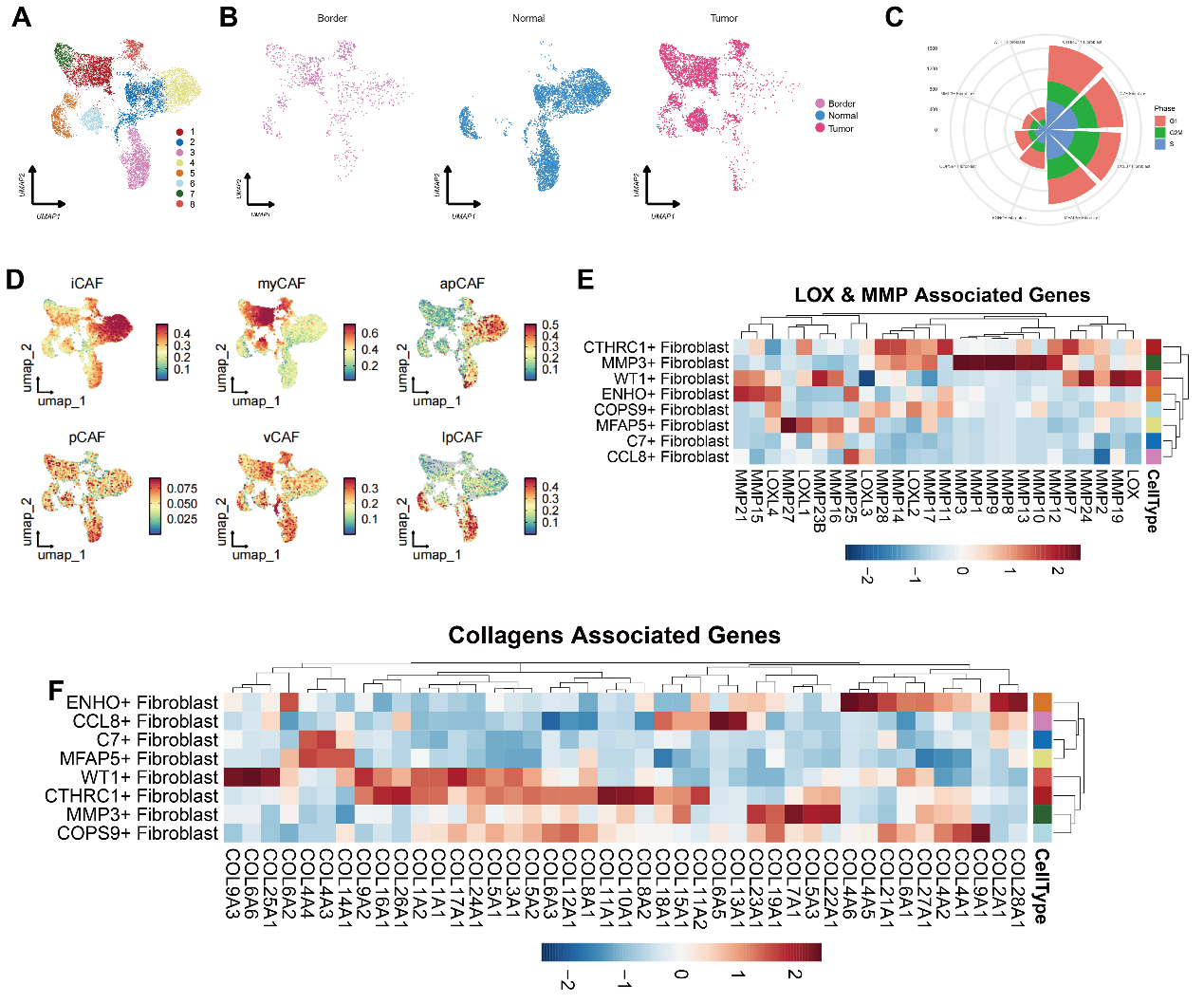


**Figure S1. The distribution characteristics and gene expression profiles of different subtypes of CAFs in CRC samples.**

(A) UMAP plot shows that CAFs are clustered into eight distinct subtypes. (B) The distribution of these CAFs in normal, border, and tumor samples. (C) The radar chart shows the distribution of cell cycle states among different CAF subtypes. (D) The UMAP plot shows the distribution of the phenotypic scores of six established subtypes among all CAFs in our study; the color scale represents the gradient of the scores. (E) The heatmap shows the average expression levels of lipoxygenase (LOX)- and matrix metalloproteinase (MMP)-related genes in different CAF subtypes. (F) The heatmap shows the average expression levels of collagen (COL)-related genes in different CAF subtypes.


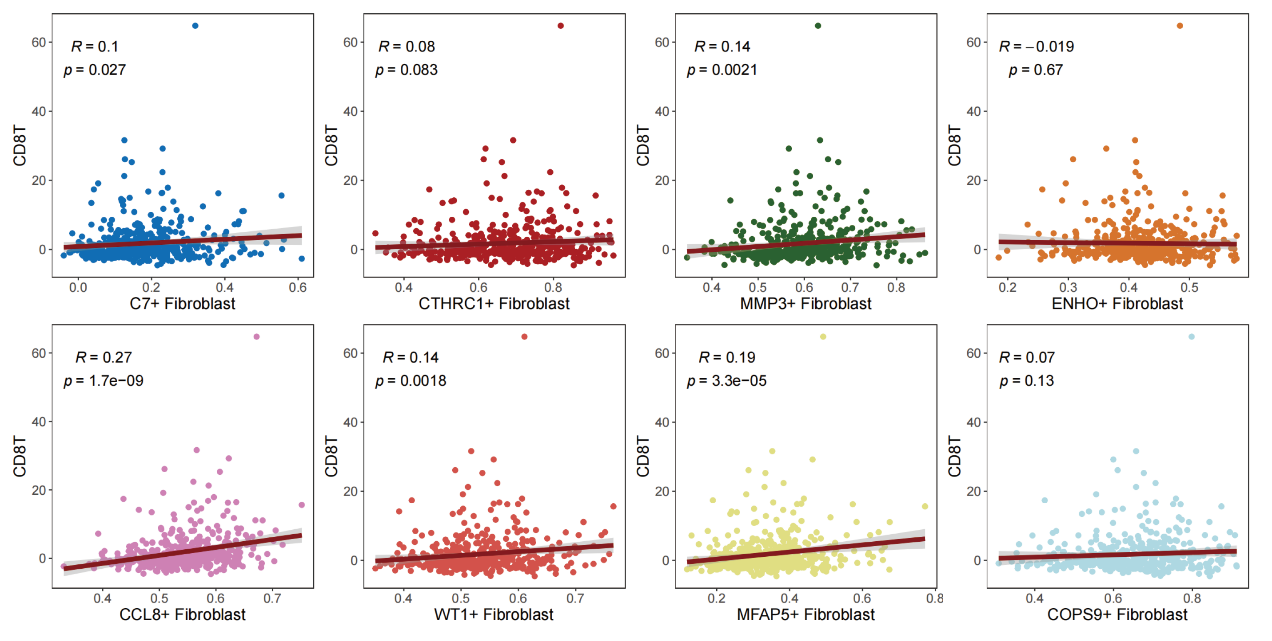


**Figure S2. The correlations between the abundance of different CAF subtypes and T cell infiltration abundance in the TCGA-COAD dataset.**


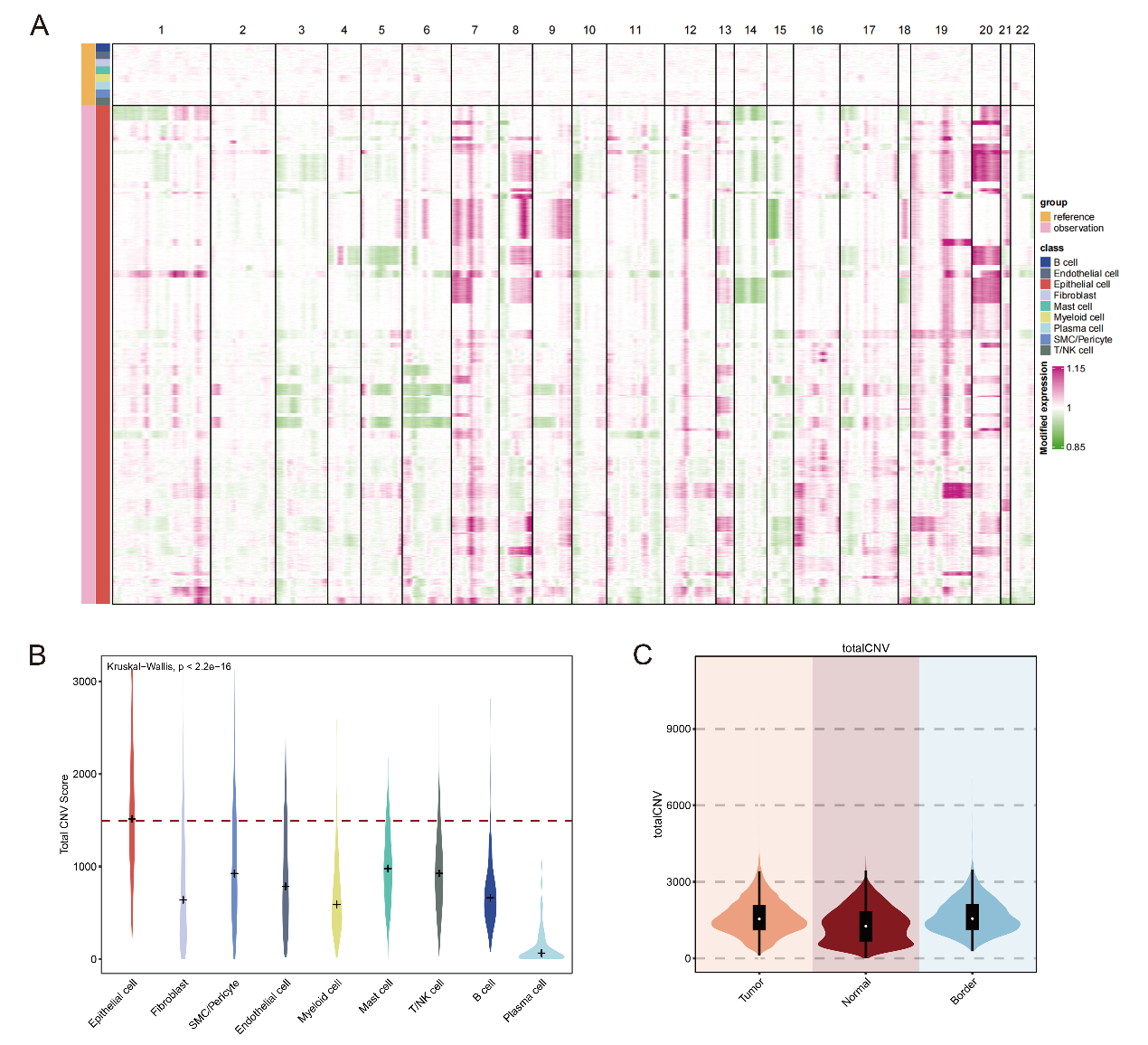


**Figure S3. CNV scores were estimated using inferCNV algorithm and were compared among different cell types.**

(A) The heatmap of inferCNV results for epithelial cells, using immune cells as a reference. (B) The violin plot of CNV scores for different cell types; compared to other cells, the CNV score of epithelial cells is the highest. (C) The violin plot of CNV scores for epithelial cells from different sources (normal, border, tumor).
